# The state of research into children with cancer across Europe: new policies for a new decade

**DOI:** 10.3332/ecancer.2011.210

**Published:** 2011-02-09

**Authors:** K Pritchard-Jones, G Lewison, S Camporesi, G Vassal, R Ladenstein, Y Benoit, JS Predojevic, J Sterba, J Stary, T Eckschlager, H Schroeder, F Doz, U Creutzig, T Klingebiel, HV Kosmidis, M Garami, R Pieters, A O’Meara, G Dini, R Riccardi, J Rascon, L Rageliene, V Calvagna, P Czauderna, JR Kowalczyk, MJ Gil-da-Costa, L Norton, F Pereira, D Janic, J Puskacova, J Jazbec, A Canete, L Hjorth, G Ljungman, T Kutluk, B Morland, M Stevens, D Walker, R Sullivan

**Affiliations:** 1 University College London, UK; 2 Evalumetrics UK and University College London, UK; 3 Kings College London, UK; 4 Institut Gustave-Roussy, Villejuif, France; 5 St. Anna Children’s Hospital, Austria; 6 Ghent University Hospital, Belgium; 7 Children’s Hospital Banja Luka, Bosnia-Herzegovina; 8 University Hospital Motol, Prague, Czech Republic; 9 Aarhus University Hospital, Skejby, Denmark; 10 Institut Curie, Paris, France; 11 Society for Pediatric Oncology and Hematology (GPOH), Münster, Germany; 12 Children’s Hospital ‘Aglaia Kyriajou’ Athens, Greece; 13 2nd Department of Paediatrics, Semmelweis University, Hungary; 14 DCOG, The Netherlands; 15 Lady’s Children Hospital, Crumlin, Ireland; 16 Istituto G. Gaslini, Italy; 17 Catholic University of the Sacred Heart, Rome, Italy; 18 Vilnius University Children’s Hospital, Lithuania; 19 Mater Dei Hospital, Msida, Malta; 20 Medical University of Gdansk, Poland; 21 Children’s University Hospital, Lublin, Poland; 22 University Hospital S. João—Porto, Portugal; 23 Instituto Português de Oncologia do Porto (IPO Porto), Portugal; 24 Portuguese Institute of Oncology, Lisbon, Portugal; 25 University of Belgrade, Serbia; 26 Division of Pediatric Hemathology and Oncology, Slovakia; 27 Children’s Hospital, University Medical Centre Ljubljana, Slovenia; 28 H. U. La Fe Pediatric Oncology Unit, Spain; 29 Paediatrics, Clinical Sciences Lund, Sweden; 30 Paediatrics, Clinical Sciences Uppsala, Sweden; 31 Hacettepe University, Turkey; 32 Birmingham Children’s Hospital, UK; 33 University of Bristol, UK; 34 University of Nottingham, UK; 35 Centre for Global OncoPolicy, Kings Health Partners Integrated Cancer Centre, London, UK

## Abstract

Overcoming childhood cancers is critically dependent on the state of research. Understanding how, with whom and what the research community is doing with childhood cancers is essential for ensuring the evidence-based policies at national and European level to support children, their families and researchers. As part of the European Union funded EUROCANCERCOMS project to study and integrate cancer communications across Europe, we have carried out new research into the state of research in childhood cancers. We are very grateful for all the support we have received from colleagues in the European paediatric oncology community, and in particular from Edel Fitzgerald and Samira Essiaf from the SIOP Europe office. This report and the evidence-based policies that arise from it come at a important junction for Europe and its Member States. They provide a timely reminder that research into childhood cancers is critical and needs sustainable long-term support.

## Executive summary and policy conclusions

1. Between the period 1997–2008, there were 31,144 research papers published by the global paediatric oncology community representing about 5% of all cancer research; the number rose rapidly during the final 3 years but was almost constant from 1997 to 2002. About one third are from the European Union (EU), one third from Canada and the USA, and one third from the Rest of the World (RoW). Paediatric oncology is a vibrant and growing research community which has delivered major advances across many childhood cancers in terms of survival and quality-of-life improvements.
2. Paediatric oncology papers are cited less (in a 5-year window) than the average for the journals in which they are published, although both actual and potential citation scores have been rising. The Netherlands’ papers are the most cited, followed by those of the USA, the UK and Sweden, and its papers are also the most likely to be in the top centiles of citation counts. The UK, the USA and Canada write the most reviews, a new measure of the esteem in which a country’s senior scientists are held. Similar indicators were also determined for the leading research institutions in North America and Europe: the former’s were the most highly ranked, led by the Dana-Farber Cancer Institute in Boston and the US National Cancer Institute in Bethesda, MD. The leading European centres were in the Netherlands (Amsterdam and Rotterdam), Germany (Munster), France (Inst. Gustave Roussy, Villejuif) and University College London.
3. Sweden and the Netherlands did the most basic paediatric oncology research (on average) but the differences between countries were not large. About 7% of the papers reported clinical trials; Switzerland did the most (almost 15% of its papers) and Spain and the USA, the least (among the countries selected for analysis). Advances in paediatric oncology depend on research activity and support across a wide spectrum of research domains from the most basic to applied. Constant assessment of this ‘balance’ and national and European levels is necessary to ensure no detrimental lacunae occur in the paediatric oncology research spectrum.
4. An analysis of countries’ and institutions’ willingness to collaborate internationally (expressed as a ratio of observed numbers of collaborative papers to those expected on the basis of countries’ percentage presence in the subject area) showed close collaboration, as expected, between Canada and the USA, but relatively little between North America and Europe. EU Member States were collaborating increasingly with each other, especially Germany and the Netherlands, and also Switzerland with France, Germany and Italy. Netherlands researchers are particularly sought after, perhaps because of their strong citation performance. Researchers from the RoW are largely ignored by the Europeans, but less so by the USA and its leading institutions. There is a case to look further at how Europe and the USA can work together more effectively. Likewise the global burden in paediatric oncology is shifting towards ‘younger’ developing countries. There is an urgent need for greater research cooperation, that is not yet present, between developed and transitional countries.
5. The funding of paediatric oncology research in the 10 selected countries was determined by the examination of the financial acknowledgements on 2575 papers from 1997–2000 and 2005–8. Funders were classified into five main sectors: national government and private-non-profit (PNP), industrial, international and other (foreign). In most European countries except Spain, PNP sources out-numbered government ones, but almost half the papers bore no acknowledgement—a marker of fragile, short-term funding. The dominant role in the USA of the National Cancer Institute and other components of the National Institutes of Health was very apparent: they supported nearly half the US total. In Sweden, collecting charities and endowed foundations each funded over 40% of the research papers. Other nationally prominent funders were the Associazione Italiana per la Ricerca sul Cancro in Italy (25% of Italian papers), the Netherlands Cancer Society and Cancer Research UK (each just under 20% of national papers). The European Commission is playing an increasing role and funded about 7% of the papers from the seven selected EU Member States in 2005–8. The enhanced support of paediatric oncology research by the EU is a positive step forward, however, the perception is that this still remains inadequate for the scale of the problem. Furthermore, at national level funding is either too low or too fragile with significant activity reliant on short-term ‘soft’ funding. National level funding needs to be more sustainable and coherent.
6. Estimates were made of the financial resources being applied to paediatric oncology research world-wide in 2008. Based on estimates of the average cost of a paper and the number of publications, together with an analysis of the ‘hidden’ costs of the pharmaceutical industry not revealed in terms of published papers, it appears that the total was about $1.23 billion (US), of which an estimated 53% was from public/federal sources ($656 million), 27% from PNP sources ($328 million) and 20% ($245 million) from industry. The low level of funding in many countries coupled to the very small contribution by the private sector is a major concern. There is a strong case for a private global fund for paediatric oncology to be established, that could support transnational collaborative research efforts that are necessary in these rare diseases.
7. Information on childhood cancers varies considerably across Europe, factors that influence this aspect of care include:
  - involvement of parent organizations
  - the use of digital media
  - the adoption of a common standard for information provision.
  There is a need for new policy proposals to tackle this diversity of access to information which could include establishment of a European Common Information Portal in order to promote harmonization, enhanced control of information quality, standards of provision and linguistic access and the tackling of major deficiencies in countries with no patient organizations and/or native language information provision.
8. In the survey of key opinion leaders in paediatric oncology from across Europe a number of key policy areas were highlighted as MAJOR issues in ensuring the future of childhood cancer research and continuing improvements in survival. There was consensus on the need for
  - Adequate EU funding to support a Europe-wide clinical trials network to assist with testing and dissemination of novel therapies and techniques. ENCCA has been an important tool in delivering solutions in this area. However, this is short term and sustainable, long-term research networks must be created and funded.
  - A reduction of EU trial bureaucracy/regulations to remove barriers to initiation and conduct of investigator-led clinical trials which could include a European Trials Bureau. Furthermore there needs to be a better understanding by regulatory policymakers of the level of risk for children with cancer participating in clinical trials (currently overestimated by insurers as well). It is essential that the EU Clinical Trials Directive (CTD) is modified if investigator-driven clinical trials are to have any future.
  - The creation of a European Parent/Survivor organization to assist with enhancing quality of patient information for childhood cancers.
  - The creation of a European Childhood Cancer Epidemiological Registry (essential for outcomes research) and linked Biobank Facility to enhance awareness of childhood cancer incidence, support development of service provision and facilitate access to linked population data and tissue samples for research.
  - EU support for enhanced harmonization of treatments through Pan-European Guidelines for treatment.

## 1 Introduction

### 1.1 Origins of study

This report has been conducted under the auspices of the EUROCANCERCOMS project as a collaboration between SIOPE and Kings Health Partners Centre for Global OncoPolicy (ECRM Foundation).

### 1.2 Data to be presented

The original written specification called for the following tasks:

1. Development of a ‘filter’ to identify papers in the Web of Science (WoS) database that are in both the subfields of paediatrics and oncology.
2. Comparison of the outputs of Europe (with division by Member State), the USA and Canada, during the 11 years 1997–2007. [This was subsequently amended to 2008 as data on this year became available.]
3. Identification of the leading research institutions in paediatric oncology in Europe, the USA and Canada.
4. Determination of the relative commitment of individual countries to paediatric oncology, compared with all oncology and with all biomedical research in the same years.
5. An analysis of the potential and actual citation impact (ACI) of the paediatric oncology papers (for different countries).
6. Comparison of papers describing phased clinical trials within the subject area to overall outputs in paediatric oncology.
7. Provision of a matrix showing the amount of collaboration between individual countries, and any changes over time.
8. Provision of a matrix showing the amount of collaboration between the leading research institutions with selected foreign countries.
9. Listing of the major funders of paediatric oncology research, with a breakdown by country and type (sector) of funding body, including industry.
10. Estimation of the relative amount of funding for paediatric oncology from public and private sources, the latter comprising both PNP and commercial.

### 1.3 Notes on the specification

This bibliometric study has been confined to documents classed as articles or reviews in the WoS, and to publication years 1997–2008. The documents were to be identified within the intersection of two sets: oncology papers (for which there was already a filter, devised in consultation with Dr Lynne Davies of Cancer Research UK) and paediatric papers. The oncology subfield was defined as:

The study and treatment of cancer or tumours. This incorporates academic oncology and clinical oncology. Academic oncology is aimed at identifying the causative agents or underlying genetic defects producing cancer and at developing these discoveries into effective drugs and other therapies. Clinical oncology is oriented towards the treatment, management and cure of cancer.

Papers in paediatrics, defined as:

Study of the causes, effects and treatments of disease or disability in infants and children were to be identified by means of a second filter, but this needed updating and extending to cover additional journals in the WoS but not in the Science Citation Index on CD-ROM for which the filter had originally been defined, and journals appearing later than 2005.

The geographical analysis has been conducted using both integer and fractional counts for countries in the addresses of the papers. [A paper with two UK addresses and one French one would count unity for each country on an integer count basis, but 0.67 and 0.33 respectively on a fractional count basis.] However, relative commitments for the individual countries have been determined using only integer counts as it was not practical to download all the world papers in oncology and biomedical research over 12 years in order to determine the values using fractional counts. For the analysis of research institutions, and of international collaboration, only fractional counts have been used, see section 2.6.

Both potential and ACI have been determined as numbers of citations in a 5-year period beginning with the year of publication. Mean citation scores for papers in the journals used (potential citation impact—PCI) were obtained from Thomson Reuters, the publishers of the WoS for every 2 years from 1996–2002; for 2004 they were obtained directly from the WoS^1^. Actual citation scores for individual papers were obtained from the WoS, but only up to 2004 publications (cited in 2004–8). An additional indicator of research quality was obtained from the numbers (and percentages) of papers from each country that were cited highly enough to be in the top centiles: this is called the ‘world-scale’ value. Finally, another indicator of merit was provided by the percentages of reviews for each country. This is a new indicator and aims to show the esteem in which senior researchers are held by journal editors who commission the reviews.

## 2 Methodology

### 2.1 Selection of papers

The papers were selected from the WoS that were in both subfields, paediatrics (PAEDI) and oncology (ONCOL). The filters used to define these two subfields are given in Annex A, where SO is the journal name and TI is a word in the title of the papers. The bibliographic details of the selected papers were downloaded to a series of individual files, each of which contained 500 papers. They were opened in succession by a macro written by Philip Roe and the papers’ parameters (authors, title, source, document type and addresses) were collected in an Excel file for analysis. This file contained data on a total of 31,144 papers.

For the comparison groups of papers in cancer research overall and in all biomedicine (BIOMED), the WoS was interrogated and the world totals and those for each of the ten selected countries (see Table 1) were determined, year by year. The biomedical papers were specified by means of an ‘address’ filter, based on cognitive words in the papers’ addresses [1,2]: this works well in distinguishing between biomedical and non-biomedical papers in multidisciplinary journals such as *Nature* and *Science*. Details are given in Annex A.

In this report, the countries are referred to by their ISO digraph codes, as shown in Table 1. In addition the output of the other 20 EU Member States as a group (EU20) was determined and used in the analysis as was that of the RoW.

### 2.2 Identification of the leading research institutions

The intention here was to find the institutions in each of the selected countries with the largest outputs over the 12-year period, based on fractional counts. This was made difficult because many institutions had alternative names, particularly universities and associated hospitals. For the USA, there were 188 different organizations listed on a special Website, for Canada, 16 on the same Website, and for the eight European countries, a total of 98 places with output >100 papers. These latter were selected as the outputs of cities unless it was clear that there were several separate institutions within the same city. The intention was not to determine the outputs of all these places, but rather of the top ones in the USA, in Canada and in Europe. Each of the 188 + 16 + 98 organizations or places were given an individual trigraph (three-letter) code, of which the first two letters were the abbreviation for the US state, Canadian province or European country. Each one was sought among the addresses on each paper using search strings chosen to include the name variants of the organization or place; one string (the name of the city and state/province/country) was required together with one or more of any others that were specified. For some European cities, ‘no’ strings were also specified in order to distinguish the outputs of two (or more) different institutions, e.g. in Amsterdam and Milan (two universities). Some examples of search strings are shown in Table 2 for the USA and Canada, and in Table 3 for European countries.

For the UK, the Institute of Cancer Research’s output could be specified with the presence of the town, ‘SUTTON’ and ‘ENGLAND’ (there is also one in Canada); for Queen Mary, University of London, which includes the Royal London Hospital and St Bartholomew’s Hospital, the search was for ‘LONDON’ and ‘ENGLAND’ and either BART or LONDON?HOSP (the ? indicates a single character) or QUEEN?MARY. For the output of University College London, eight possible strings were needed in addition to LONDON and ENGLAND.

### 2.3 Determination of relative commitment to paediatric oncology

This parameter is the ratio of a country’s percentage presence in paediatric oncology to its percentage presence in all oncology, or in biomedical research. In order to keep the amount of data to a reasonable level, these ratios were calculated for three 4-year periods, 1997–2000, 2001–4 and 2005–8. For example, in 2005–8, the UK published 934 paediatric oncology papers out of a world total of 12,203 or 7.65%, whereas in cancer research as a whole it published 15,616 out of 214,053 or 7.29%. Therefore its relative commitment (RC) was 7.65/7.29 = 1.05.

### 2.4 Potential and ACI

Journals can be characterized by the average number of citations to papers published in them in a given year and received in a given time window. Because the peak year for citations is usually the second or third year after publication, we have taken a 5-year window (i.e. the year of publication and four subsequent years). The PCI of a paper is then the expected number of citations that it would receive if it were an ‘average’ paper, i.e. the total number of citations divided by the number of papers (Table 4).

The ACI was determined directly from the WoS, with data for citations, year by year, being downloaded 500 at a time to separate files. These were processed by another macro written by Philip Roe so as to provide a single spreadsheet, with re-creation of the bibliographic source (journal, year, volume, issue, pages) for each paper that could then be matched to the source in the main file of papers. The numbers of citations in the 5-year window could then be copied across to the main file for each paper. However, for a few papers, the source was not unique (this applied particularly to the new all-electronic journals) and a match had to be made on the paper title. Because a 5-year window was used, the values of ACI were only available for papers published from 1997–2004 (8 years).

We also determined how many of a country’s papers were cited highly enough to put them in the top 5% of the world (31 cites in 5 years), top 10% (21 cites) or top 20% (12 cites). These three percentages, when compared with the world values^2^, multiplied by 100 and averaged, are called ‘world-scale’ values (by analogy with oil tanker charter rates) [3].

Another measure of merit, or esteem, is the percentage of reviews in a large set of papers. Since these are usually invited from distinguished scientists, their presence does provide an additional measure of how well these are regarded by the editors of journals [4]. This measure needs to be normalized relative to the world mean value and this can be done with respect to both paediatric and to all cancer research. The ratios show how well senior paediatric oncology researchers from different countries were regarded and they can also be compared with the esteem of their oncology researchers overall.

### 2.5 Types of research: clinical or basic, and phased clinical trials

Biomedical research papers can be classified on a scale from 1 = clinical to 4 = basic, both in terms of the journal in which they are published (RL_j_), and (for a group of, say, 20 or more) in terms of their individual titles (RL_p_). The classification system described in reference 2 was used to characterize them (Table 5).

A special macro was used to classify all the paper titles in the file as containing either one or more ‘clinical’ words, or one or more ‘basic’ words, or both. For a group of papers, the mean RL was then calculated, with each paper with a clinical title word being counted only as 1.0, each paper with a basic title word only as 4.0, and each paper with both as 2.5. [However, about 14% of the papers could not be classified in this way.] This showed whether papers from a given country were being published in relatively clinical or relatively basic journals, compared with their subject matter. It could also show if the papers were becoming more clinical or more basic with time.

In order to identify papers concerned with clinical trials, their titles were filtered to show which ones contained any of the words, ‘double blind’, ‘phase’, ‘study’ or ‘trial’^3^. The total was 2307, or 7.2% of the total. These papers were then analysed by country and by year, to show any time trends. Relatively few of these papers contained the word ‘phase’, which is associated with the clinical trials of new drugs.

### 2.6 International collaboration for countries and institutions

It is well known that the amount of international collaboration in research has been steadily increasing with time, and it is also more common in basic research than in clinical work. Thus for the UK papers in paediatric oncology, 32% of those with RL_j_ < 1.5 had foreign coauthors, but the percentage rose to 37% for RL_j_ between 1.5 and 2.0, 46% for RL_j_ between 2.0 and 3.0 and as much as 61% where RL_j_ exceeded 3.0. Any comparisons of rates of international collaboration must therefore take account of both time and RL, as well as national factors such as possession of a common language or geographical proximity.

The standard method of presentation of collaboration data is with the Salton Index (SI), equal to the number of papers co-authored by the two entities divided by the square root of the product of the two individual totals. This is non-dimensional, and is usually expressed as a percentage. However it is unsatisfactory because the index depends on the size of the geographical entities and can give misleading results if they are very different. Instead, we have determined the amount of collaboration by a different method, illustrated by means of an example.

From the point of view of a given country, say Canada, its papers have a foreign contribution divided up between other countries on a fractional count basis, with each country providing a certain percentage of the total. Thus the 1556 Canadian papers in paediatric oncology had a fractional Canadian contribution of 1086 papers and a foreign contribution of 470 papers. Of these, the USA contributed 305 and Germany 21 papers, both on a fractional count basis, i.e. 64.9% and 4.5% of the foreign total. But the USA’s fractional contribution to paediatric oncology was 31.0% and Germany’s was 7.4%, and if we correct these figures to exclude Canadian papers from the world total, they increase slightly to 32.1% and 7.7%, So the USA was *over*-selected by Canadian researchers by a factor of 64.9/32.1 = 2.02 and Germany was *under*-selected as a partner by 4.5/7.7 = 0.58. On the basis of the two countries’ presence in world paediatric research *minus* that of Canada, we would have expected 151 US papers and 36 German ones. The difference for the US contribution is highly statistically significant; for the German contribution it is significant at *p* = 1%.

A rather similar approach was used for the calculation of the tendency of individual research institutions to collaborate with foreign countries. Their presence was determined in each paper on a fractional count basis, and for the selected institutions (the ones with the largest output in the USA, Canada and Europe) we calculated the contribution made by other institutions in their own country, and by the other nine selected countries, EU20 and the RoW. These percentages could then be compared with their respective presences in paediatric oncology. An allowance was made for the contribution of the institution being considered even though such a correction was very small. The calculations were made by a special macro, written by Philip Roe, which ran for over 9 minutes and analysed the 102,922 addresses on the 30,944 records with addresses.

### 2.7 The funding of paediatric oncology research

The purpose of this part of the study was two-fold: to show which organizations were funding the research, and to estimate the resources being devoted to the subject area. For the first task, samples of papers from each of the ten selected countries in each of the two 4-year periods, 1997–2000 and 2005–8, were looked up in the British Library and other London libraries, either online or by inspection of the printed documents. The sample sizes were proportional to the square root of the numbers of papers, with a minimum of 60, see Table 6, except for the UK in 1997–2000 for which funding data had previously been obtained as part of the Research Outputs Database [5], a project of The Wellcome Trust from 1993–2003. The sample sizes were designed to give a total of 1250 papers in each of the two quadrennia.

The funding bodies acknowledged, either explicitly or implicitly through the addresses on the papers, were recorded as three codes: one (a trigraph) for the individual funding body, e.g. MRC = Medical Research Council, NCI = (US) National Cancer Institute; the second, the ISO digraph country code; and the third, another digraph denoting the sector and sub-sector of the funding body, as shown in Table 7.

Implicit funding, derived from addresses, is assumed for government agencies, collecting charities and industry, but not usually for foundations, which often give their name to buildings but do not necessarily fund the research.

It is to be expected that a large minority of the papers will not have any funding acknowledgements, either explicit or implicit. Most such research will in practice have been supported, at least in Europe and in Canada, by state-funded universities and hospitals, but these are not specifically credited unless there is an explicit acknowledgement indicating that there was a formal decision to support the research.

**Figure 1:**
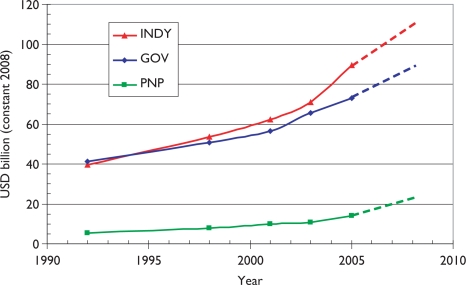
Estimates of global expenditure on health research, 1992–2005, extrapolated to 2008 (constant 2008 USD). GOV = public sector; PNP = private-non-profit; INDY = commercial

We have developed a methodology [6,7] for the estimation of the global resources being applied to biomedical research in a given area, based on bibliometric data. This involves an estimate of the average cost of a paper, which is then multiplied by the number of papers published in a year. Corrections are needed for the additional (hidden) costs incurred by pharmaceutical companies in the support of confidential research that is not published, and some smaller adjustments may be needed to allow for the varying costs of research in different countries. Triangulation is possible with reference to the global total resources being applied to health research, as estimated by the Global Forum for Health Research; see Figure 1.

The estimated total expenditure for 2008 is about $110 billion from industry, $90 billion from the public sector (including international sources, which derive largely from governments), and just over $20 billion from the PNP sector, total about $220 billion. In that year, the WoS recorded close to 480,000 biomedical research papers (articles and reviews), making the mean cost per paper about $460,000. But much of the commercial expenditure would not have resulted in publications, and in practice only about 15% of the output was supported by industry, leaving about 410,000 papers supported by $110 billion of public and PNP funds, at a mean cost of $268,000. [This corresponds to $221,000 in 2001, close to^4^ the estimated cost of a cancer paper in that year, based on a survey of researchers, of $232,000^6^.] For 2008, it is probably fair to assume an average cost for paediatric oncology papers of about $280,000. To this cost should be added the ‘hidden’ costs borne by pharmaceutical companies, discussed in more detail in section 3.10.

## 3 Results

### 3.1 Outputs of papers in paediatric oncology

**Figure 2:**
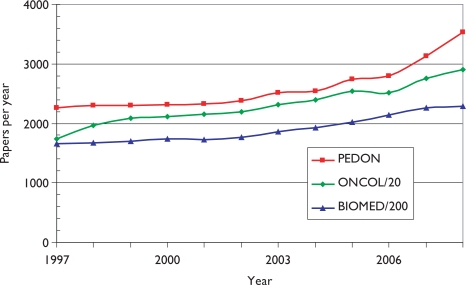
Outputs of papers (articles + reviews) in the Web of Science, 1997–2008 (publication years), for paediatric oncology (PEDON), oncology (/20) and biomedical and health research (/200).

Figure 2 shows the numbers of papers in paediatric oncology (PEDON), year by year, over the 12-year period, with for comparison (scaled) values for oncology overall (ONCOL) and for biomedical and health research (BIOMED).

Paediatric oncology represents just over 5% of all oncology research papers, which themselves account for 12% of all biomedical and health research during the period, and although the output was rather constant in the late 1990s, it has grown quite rapidly in the last 2 years.

**Figure 3:**
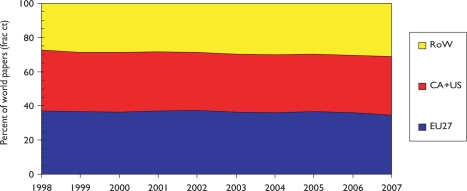
Outputs of paediatric oncology papers from three world regions, 3-year running means, fractional counts (frac ct) expressed as percentages of world total.

The outputs of individual countries are shown in Table 8 on a fractional count basis for the three 4-year periods. Although most countries have increased their absolute level of output, for most their percentage presence in the world total has decreased, because of the rapid rise in output from China and some other Far East countries. Overall, about one third of world output comes from the EU, one third from the USA and Canada, and one third from the RoW, see Figure 3.

**Figure 4:**
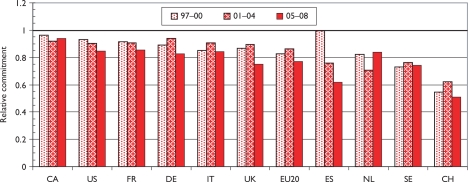
Relative commitment of 10 selected countries to paediatric oncology research within oncology, 1997–2008 (articles + reviews in the WoS).

The relative commitment of the selected countries to paediatric oncology, compared with their presence in oncology research, is shown in chart form in Figure 4. Perhaps surprisingly, this is below unity for all the selected countries—this means that it must be above unity for countries in the RoW, mostly middle-income developing countries with a large youthful population. For example, Turkey had a RC of over 3, and both India and Brazil had RCs over 1.5, during the last 2 years. Within Europe, Switzerland has much the lowest RC, followed by Spain, whose RC has reduced sharply over the study period.

### 3.2 Leading research institutions in selected countries

Application of the special macro (see section 2.2) to the set of addresses given, for each paper, the fractional count of each of the named institutions or (for Europe) the cities or universities selected for analysis. There were 16 US institutions with a fractional count of at least 100 papers over the 12-year period, and one Canadian one (the Hospital for Sick Children in Toronto); Table 9 lists them in descending order of output. In the eight selected European countries, there were 20 cities or universities that had at least 100 papers, and they are listed in Table 10. The two tables show by tinting those institutions or cities that more than doubled their output between 1997–2000 and 2005–8 (bright green), those that increased output by a factor of 1.41 (light green), and those whose output actually went down (yellow).

Although more European cities/universities/institutes met the 100 paper criterion for inclusion in the list than North American institutions, the leading European centre (University College London, which incorporates the Institute of Child Health and Great Ormond Street Hospital) would only rank eighth on the North American list, and the leading US institution, St Jude’s Children’s Hospital in Memphis, TN, has more than twice University College London’s output of paediatric oncology papers. Several European cities have actually reduced their output between 1997–2000 and 2005–8, including two each in Germany and Spain. The reduction in Spanish output from 1997–2000 was seen in Table 8 and also in Figure 4.

### 3.3 Potential and ACI

**Figure 5:**
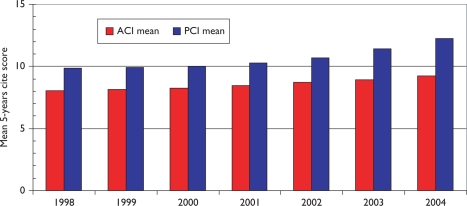
Mean potential citation impact (PCI) and actual citation impact (ACI) scores for paediatric oncology papers, 1997–2005 (3-year running means); citation window 5 years.

**Figure 6:**
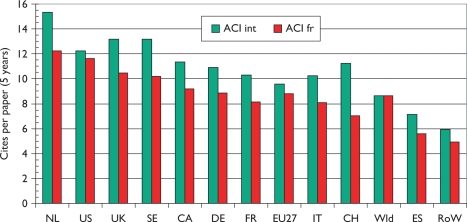
Mean value of actual citation impact (ACI) for paediatric oncology papers from selected nations, 1997–2004, on an integer count basis (green bars) and fractional count basis (red bars).

Over the 12-year period of the study, there has been a tendency for the mean citation scores of papers to increase. Figure 5 shows this effect for both PCI scores and those for ACI; it is also apparent that the latter are uniformly lower than the former, showing that paediatric oncology papers receive fewer citations than the average for the journals in which they are published. [This is not the case for cancer papers in general.] There are differences between countries in terms of the mean values of these indicators, see Figure 6, where mean ACI is shown for the selected nations on both an integer and a fractional count basis. The latter is always lower than the former, especially for small countries, many of whose papers will be internationally co-authored. All the selected countries except for Spain perform better than the world average, which is reduced by the low citation scores achieved by papers from the RoW.

**Figure 7:**
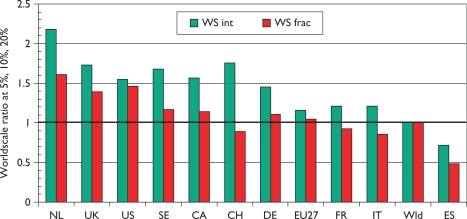
Mean world-scale values for papers in the top-cited 5% (with 31 cites in 5 years), 10% (21 cites) and 20% (12 cites) of paediatric oncology papers, 1997–2004, from selected countries.

Figure 7, which shows mean world-scale values, tells a rather similar story to that of Figure 6, with the Netherlands again scoring best, followed by the UK and the USA, and Spain again scoring the least.

**Figure 8:**
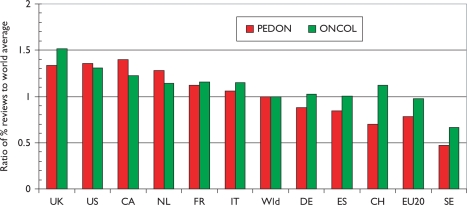
Percentages of reviews for selected countries, 1997–2008, relative to the world mean, in paediatric oncology papers (PEDON) and in all cancer research (ONCOL).

**Figure 9:**
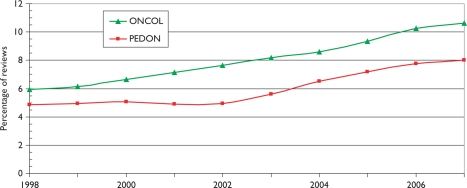
World mean percentage of reviews (of total of articles and reviews) in paediatric oncology papers (PEDON) and all cancer research (ONCOL), 1997–2008, 3-year running means.

Another measure of esteem is the percentage of reviews that countries write. In Figure 8 the percentages relative to the world mean have been shown both for paediatric oncology and for all cancer in the same years. The world mean percentage for paediatric oncology was 5% up to 2002, and then rose almost linearly to 8% in 2007–8; this was somewhat lower than the corresponding figures for all cancer research, Figure 9.

### 3.4 Citation scores and percentage of reviews for institutions

For each of the institutions with more than 100 papers (on a fractional count basis) listed in Tables 9 and 10, three measures of impact or esteem were determined and the institutions are listed in descending order of mean ‘quality’ of output, shown as ratios to the world mean in Tables 11 (for North America) and 12 (for Europe). The cells are tinted to show those with values > 2.0 (bright green), > 1.41 (pale green), < 0.71 (pale yellow) and < 0.5 (pink) (Table 11).

Six of the US institutions, and one European university, have actual citation scores more than twice the world average. All the US institutions wrote more reviews than the world average, but 11 of the 20 European cities/universities wrote fewer, possibly because of language bias. However the relatively poor showing of the Karolinska Institutet on this measure is rather surprising, particularly as it scores well on the two citation measures (Table 12).

### 3.5 Research level and participation in clinical trials

**Figure 10:**
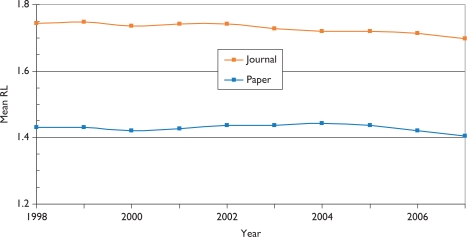
Mean research level (1 = clinical, 4 = basic) for paediatric oncology papers, 1997–2008, 3-year running means, based on paper titles (Paper) and the journals in which they appeared (Journal)

Figure 10 shows the variation in research level (RL) with time based on both the actual titles of the paediatric oncology papers and the journals in which they were published. It is immediately apparent that the papers are much more clinical than the journals in which they appear, so that the researchers are clearly aiming at a more general readership, and also to publish in higher impact journals than their titles would suggest, perhaps because more basic journals normally receive more citations than do clinical ones. This is consistent with Figure 5, in which it appeared that the paediatric oncology papers were less cited, on average, than the papers in the journals in which they were published. There is a slow decline in the mean value of RL_j_ (i.e. the journals used for paediatric oncology papers are becoming a little more clinical), but not in that of RL_p_.

**Figure 11:**
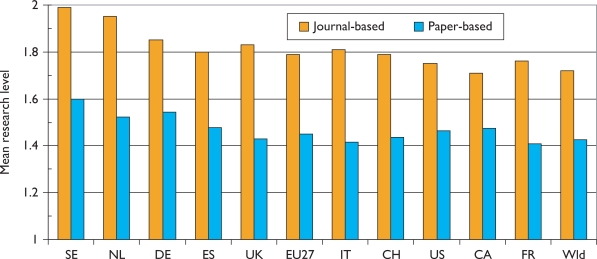
Mean journal-based and paper-based research levels for selected countries in paediatric oncology, 1997–2008; integer counts.

There are some differences in the level of research carried out in the selected countries. This is shown in chart form in Figure 11. Sweden and the Netherlands do the most basic research, and Canada and France the most clinical, but the differences are not great.

**Figure 12:**
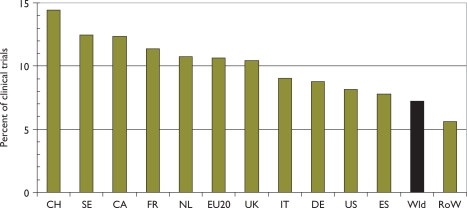
Percentage of selected countries’ paediatric oncology papers that are clinical trials, 1997–2008; integer counts.

There were 2239 papers reporting clinical trials, according to the definition described in section 2.5; the percentage rose from about 6.5% to 7.5% over the period but somewhat irregularly, with a temporary peak in 2001 and a small trough in 2005. There was, however, a much bigger variation between countries, see Figure 12, with Switzerland performing relatively the most—almost twice the world average of 7%—and the RoW rather few.

### 3.6 International collaboration for countries

The matrix of inter-country collaboration is not symmetrical when the contributions are determined on a fractional count basis. For example, the Canadian contribution to US papers is equal to the US contribution to Canadian papers on a simple integer count basis (514 papers), but the two are not equal on a fractional count basis: they are respectively 166 and 305 papers. Table 13 shows the numbers of foreign contributions from the ‘guests’ listed in the columns to the papers by the ‘hosts’ in the rows.

These contributions were then compared with the numbers expected on the basis of each country’s (or the EU20’s or RoW’s) percentage presence in world paediatric oncology research (less that of the country in the row) to give a ratio of observed to expected numbers of papers, shown in Table 14. In this table, cells are coloured green if the ratio > 2.0; pale green if it >1.41; yellow if it <0.71 and pink if it <0.5.

This table reveals some collaboration patterns that are well known and easily explicable on the basis of geographical proximity, or historical, linguistic or cultural factors. Thus Canada and the USA collaborate well in both directions. Within Europe, the US scientists favour Switzerland and Sweden in relation to their output, but Canada favours Switzerland and the UK. The UK favours the Netherlands and Sweden, followed by France, Switzerland and the EU20 Member States, but, perhaps surprisingly, not the USA. The Netherlands has close links both with Germany and the EU20, but Germany strongly favours Switzerland, as do France and Italy, probably because of linguistic ties. The table reveals a strong intra-European linkage, stimulated by EU activities in research, with a comparative neglect of the USA and Canada, apart from above-average Canadian contribution to UK papers.

But not all the high (or low) ratios are statistically significant as some of the numbers are quite small. Table 15 shows which are significant at the 5% and 1% level: differences that are statistically significant with *p* < 1% are shown shaded in bright green (if greater than expected) or in pink (if less); if *p* < 5%, differences are shown shaded in light green (if higher) and in light yellow (if lower).

It is clear that paediatric oncology researchers from the RoW are not collaborating with the selected countries as much as their overall presence in the subject would appear to justify. This may be due to ignorance of their work, particularly if they are from the Far East. The USA is also relatively neglected, except by Italy and Spain; this is rather more surprising. Canada, on the other hand, is not significantly neglected. This table reinforces the view that the Europeans are working well together.

### 3.7 Changes in collaboration pattern with time

The above exercise was repeated for each of the 4-year periods, 1997–2000, 2001–4 and 2005–8. For many of the pairs of countries the numbers of co-publications were too small to make determination of time trends reliable, but the table below shows where the numbers are likely to be significant. Not all of the triplets of ratios of observed to expected numbers of papers (cf. Table 14) show a uniform increase or decrease, however, and the colour coding of the cells in Table 16 is intended to show the main features of the changes.

Since the countries are arranged in descending order of outputs (except for the RoW), it is not surprising that the coloured cells are mainly in the upper left quadrant. For the USA, there is an increased preference for working with German and Swedish researchers, but a decreased preference for Canadians and those from the EU20—mainly the new accession Member States. There is clearly a mutually increasing trend for British and German researchers to collaborate, and for British researchers to contribute more to Dutch papers (but not vice versa). The EU20 Member State researchers seem to be less popular in many countries, but on the other hand, they are increasingly receptive of Swedish collaborators. The other notable trend, and it is the biggest in terms of ratio change (from ×2.7 to ×5.1) is the increasing German contribution to Swiss paediatric oncology.

### 3.8 International collaboration for leading institutions

For each of the institutions (or cities/universities for Europe) listed in Tables 9 and 10, we determined their relative propensity to collaborate with researchers from the other nine selected countries, the EU20 Member States and the RoW. The methodology is explained in the last paragraph of section 2.6. The results, in the form of observed to expected ratios, are shown in Tables 17 (for North America) and 18 (for Europe).

All of the leading US institutions preferentially collaborate with Canada, as might be expected from Table 14 where the ratio for the whole country is ×2.81. Most also collaborate highly with Switzerland (except notably the University of Minnesota, MNU), and with the Netherlands. However Spanish researchers are hardly used, except by Memorial Sloan Kettering in New York (NYL) and the University of Pittsburgh, Pennsylvania (PAG). Collaboration with other countries appears to be on an individual basis with no clear pattern.

The collaborative patterns of the European cities and universities are rather more consistent than for the North American institutions. The Netherlands, in keeping with its superior citation performance (see Figure 6) is universally popular as a collaborator, as is the UK (except for the Humboldt University/Charité Hospital in Berlin, DEB) and as are the EU20 Member States. Switzerland is popular with the German, French and Italian cities and universities (cf. Table 14), despite an average citation performance; its extensive collaboration with places in these countries is stimulated by its strong showing in clinical trials. Thus of the Swiss domestic papers (*n* = 199), only 7 involve clinical trials whereas 64 of its 295 international papers do (22%), and the percentages of papers co-authored with Germans, Italians and French authors that involve clinical trials are 26%, 29% and 30%, respectively. On the other hand, US researchers are relatively little used by almost everyone, and Canadians are also relatively neglected except in the UK, Istituto Gaslini in Genova (ITG), Munster (DES) and Hannover (DEV) in Germany. Also, researchers in the RoW are resolutely ignored as potential partners, much more than by the North Americans. This suggests that the major European research places in paediatric oncology are increasingly turning towards other European countries for their partners, no doubt stimulated by EU funding, see below.

### 3.9 The funding of paediatric oncology research: acknowledgements

Not quite all of the papers scheduled to be looked up could, in fact, be found—some held in the British Library had been removed for binding, and some journals were not taken either there or in the library of University College London to which we also had access. However it was possible to determine the funding on a total of 1968 papers, plus the 607 UK ones that had earlier been processed for the research outputs database (ROD). The analysis of funding bodies was carried out separately on the papers from each of the ten selected countries by means of a macro (written by Judit Bar-Ilan) that counted the numbers of funding bodies with each of the following codes (see Table 7 for their definition):

- for the country of concern, -GA, -GD, -LA = GOV; -CH, -FO, -HT, -MI, -NP = PNP
- for all countries, -BT, -IN, -IP, -SN, -SP = INDY; -EU-, -XN- = INTL
- and, by difference from F, the number of funders, OTHER.

For each of the countries, the leading funding organizations of paediatric oncology research were also determined, with the aid of yet another macro written by Philip Roe. Because the samples of papers were of varying sizes, all the results are given as percentages in Table 19.

There are no great differences between the two quadrennia, but it appears that industry in Europe (but not in the USA) has been reducing its support for the subject, and international bodies have been increasing it in Europe except for Spain. Governmental support in Europe has also been declining except in France. In most European countries, apart from Spain, national PNP sources provide more support than does government; this is particularly noticeable in the Netherlands, and in Sweden where PNP sources support more than half the papers. Overall, almost half the papers report no specific funding (even allowing for implicit funding based on addresses) except in Sweden where the percentage is less than one fifth.

**Figure 13:**
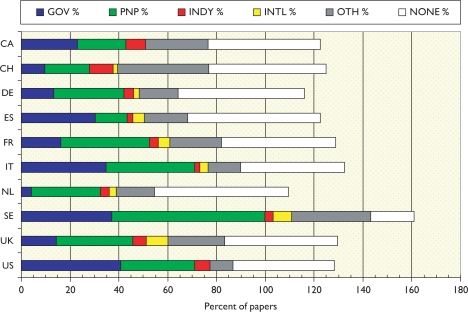
Chart showing the mean percentage support for paediatric oncology research from different funding sectors in ten selected countries, 1997–2000 and 2005–8. GOV = national government; PNP = national private-non-profit; INDY = industry; INTL = international; OTH = other. *Note: percentages add to more than 100% because of multiple funders on some papers.*

The mean percentage values are shown in Figure 13. This shows the big role played by government in the USA, and also in Sweden, Italy and Spain, but not in the other European countries.

It is of interest to sub-divide the main sectors in order to see if there are noticeable differences between the amount of support from local and regional authorities, collecting charities and endowed foundations, biotech companies and the EU. The numbers of papers in the two quadrennia together acknowledging each of these sub-sectors are shown in Table 20.

Of the EU Member States, Sweden and Spain show the most support from provincial authorities, and the Netherlands and UK the least (none). Collecting charities provide much more support than foundations except in Sweden and Spain, though the latter has only a small charitable sector. Support from biotech companies is confined to Canada, the UK and the USA, but is barely 1% of the research output. However the EU is clearly active in supporting paediatric oncology research in the EU Member States, with, on average, 4.4% of papers acknowledging this form of support. If this applied to all 11,277 papers from all the EU Member States, it would indicate that almost 500 papers in the subject area were supported by the EU. However, the most important finding is the relative lack of stable, long-term funding for paediatric oncology.

Finally, we identified the leading funders in each of the ten selected countries—either national government, national PNP organizations, or industry. Table 21 lists those organizations acknowledged, explicitly or implicitly, on at least 2% of each country’s papers. We found a large number of funding bodies, especially in the PNP sector, that were not listed in our thesaurus (which currently contains over 15,000 entries) and these were given ‘generic’ codes showing the country, sector and sub-sector so that the funders could be analysed as a group, for example ‘miscellaneous Canadian endowed foundations’. The US National Institutes of Health were sometimes acknowledged simply as ‘NIH’ and sometimes as the individual institute within NIH; the latter have all been grouped as NIH except for the National Cancer Institute, whose total may be much larger than that shown as some of the NIH acknowledgements would have been to the NCI.

### 3.10 The funding of paediatric oncology research: resources

We attempted to estimate the financial resources being applied to paediatric oncology research world-wide using the bibliometric methods described in references^6^ and^7^. Data are all for 2008. In that year, there were 3530 papers, and the average cost per paper can be estimated (see section 2.7) at $280,000. Multiplication of these two figures gives an estimated total cost of $990 million. To this must be added the ‘hidden’ costs incurred by the pharmaceutical companies.

We examined the addresses of the paediatric oncology papers for the presence of one (or more) of 21 leading pharmaceutical companies, listed in Table 22 with the search terms used. Many of the companies had recently merged or taken over other firms, and so the search terms incorporate the names of their subsidiaries, etc. We also sought the numbers of papers in the WoS for the same 12 years, 1997–2008, from the companies, and the ratio between them, i.e. the percentage of their papers that were in the subject area.

Perhaps surprisingly, the percentage of paediatric oncology papers in the companies’ portfolios does not vary greatly; the average is 0.23% and the standard error of the mean is 0.026%. We calculate therefore that paediatric oncology represents 0.23% of total commercial research expenditure in 2008, or $245 million. But in that year there were 21 papers from one of the 21 companies listed above, whose total cost is estimated at $0.28 million × 21 = $6 million, so the excess expenditure by companies, not accounted for by published output, is about $239 million. When added to the $990 million, the estimate of the cost of the published papers, we get an overall total of $1.23 billion. One fifth of this total is commercial and the rest is divided between public and PNP sources.

What proportion should be attributed to these two sources? In Europe, the papers with no funding would in practice have been supported by state funding of universities and hospitals, so the mean public contribution would have been 20% + 45% = 65%, with the PNP contribution 32% on average. To the public share we should also add the 5% of international funding (mainly the European Commission) and a little less than half of the ‘other’ funding, say 10%, to give a total of 80%. The PNP share would have been 32% + 12% = 44%. The public share was therefore 80/124 = 65%. In North America, the public contribution averaged 39% (allowing for US output being about ten times that of Canada), plus 5% to allow for the public funding of Canadian papers without acknowledgements, plus, say, 8% from the ‘other’ sector (probably mainly European), total 52%. The PNP contribution was 28% + 4% from the ‘other’ sector, or 32%. Therefore public funding was 52/84 = 62% of the total. These two figures are in good agreement, and it is probably fair to assume that, allowing for the known greater role of public funding in the RoW, the overall proportion would be about two thirds, i.e. $656 million, and the PNP sector would have contributed one third, i.e. $328 million (after deducting $6 m for the cost of papers involving industry). We therefore conclude that the division of funding world-wide for paediatric oncology research in 2008 was approximately as shown in Table 23.

## 4 The state of paediatric oncology by country

### 4.1 Summary of findings from key opinion leaders

Over a 6-week period in 2010 a survey was conducted through the offices of SIOPE of ‘key opinion leaders’ working in the field of paediatric oncology across Europe to determine their subjective views on the state of paediatric oncology at both national and European level through a framed questionnaire. Six questions were posed to these national experts, the responses of which are outlined below.

### 4.2 How is paediatric oncology delivered across Europe?

- It varies substantially, from countries which do not have enough funding for adequate provision of care, like Bosnia-Herzegovina or Serbia (where everything is orientated to adult cancer patients), to countries which have adequate care but not adequate research, to centres of excellence like Sweden, that have a completely different set of issues such as the lack experienced home staff (manpower thus has to be sourced from abroad, thereby depleting other countries of trained staff).
- Paediatric Oncology is often mixed Paediatric Haematology and Oncology and in some countries the distinction from adult oncology is not so straightforward. For example, Belgium and Spain have mixed Paediatric Hematology Oncology (PHO) Units. Not all countries specify this point, so it is unclear how widespread this mixed approach is. It is usual for all children with cancer to be treated in common units, though in some countries there are separate centres specialized in childhood solid tumours or in childhood leukaemias.
- The age cut-off for paediatric oncology and treatment in specialized paediatric oncology centres in European countries varies considerably, varying between 15, 16 or 18 years according to the responses received. Moreover, even within a country, the cut-off point for paediatric oncology treatment varies, as in the case of Portugal.
- ‘Shared care’—i.e. whether due to travel distances involved and need for urgent care, many specialist centres have established relationships with local hospitals to deliver ‘shared care’.

### 4.3 What are the key issues in paediatric oncology care & research?

- The key challenge for all countries whatever their size, is the rarity and complexity of childhood cancer. This means that all countries have a need for specialist centres to provide the necessary expertise but which means patients need to travel. For some very small countries, their population base, and hence numbers of patients in some very rare cancer types, is insufficient for them to be able to justify having capacity to deliver all complex treatments.
- A common problem for small countries (i.e. almost all countries in Europe) is the low number of cases per year (common range: 50–300) which makes implementing clinical trials on a national basis almost impossible. Thus international clinical trials have had to be founded and many respondents discussed the negative aspects of organizing such trials since the implementation of the EU CTD (2001/20/EC)
- Many national experts complained about the difficulties of running clinical trials under the EU CTD, in particular the responses received from Austria, Ireland, Poland and Slovenia. The consequences of the CTD varies considerably; what was particularly striking from the surveys was the Polish report that as a result of the EU CTD, the paediatric oncology community has been unable to activate a single clinical trial since 2007.
- Several national experts report on the challenges of prescribing drugs, in particular off-label or non-licenced drugs. Participants from Austria and Czech Republic in particular underlined this problem.
- A number of countries believe they would benefit from centralizing/harmonizing paediatric oncology treatment, care and research. For example, Belgium currently has such projects in development. Italy faces the issue of too many centres with expertise currently too decentralized; indeed Italian paediatric oncologists often have centres with less than 50 patients which is costly and difficult to create the optimal treatment and standards of care required for patients.
- The response on the Portuguese situation suggested that a greater harmonization of treatment protocols across the country would be beneficial.
- Responding to the situation in the UK, respondents suggested that a harmonization of rules on funding and clinical trial implementation across England, Scotland, Wales and Ireland would be hugely beneficial.

### 4.4 What are the sources of funding for paediatric oncology?

- Sources of funding vary substantially among the reports received, but there is one common thread: little or negligible involvement of commercial funding. Industry does not have an interest in running clinical trials for rare diseases such as paediatric tumours. Although local representatives might wish to support this activity, the decision of the big pharmaceutical companies are not taken at this level.
- Countries which benefit mainly from public governmental funds include France, Belgium (but also the European Organisation for Research and Treatment of Cancer (EORTC)), Germany, Poland, Spain, Greece, Ireland, Lithuania, Portugal.
- Countries which benefit mainly from charity organization funds and/or donations: Austria, Denmark, Sweden, UK (in particular funds received from Cancer Research UK).
- Countries that benefit from European and/or international grants include the Czech Republic and Hungary.
- Countries with a mixed system of funding include Hungary (governmental allocation of funds and grants), Italy (governmental funding and charities), the Netherlands (charities and governmental), Slovenia (governmental funds and charities), Slovakia (governmental and grants) and Turkey (governmental and grants).
- From the responses received only one country report specified pharmaceutical funding, that of the report from the paediatric oncology expert based in Malta.
- A lack of funding resources was also clearly apparent from the reports from experts in Bosnia-Herzegovina and Serbia.

### 4.5 What is the state of patient information for paediatric oncology?

- The availability of patient information varies substantially, from a very low level to a level of excellence. Common threads in the survey reports received include
  - a substantial involvement of parental organizations
  - the increasing role of digital publications like websites, blogs, patient forums etc. For example in Denmark, the Danish Childhood Cancer Foundation has created a an important resource for patients, http://danishcancer.blogspot.com/
  - a lack of common standards in Europe. Currently it is unclear for national experts as to who provides patient information. Does this need to be communicated by doctors, nurses, psychologists or all of the care team? The process and system of patient information, communication and dialogue is currently vague and uncertain. Moreover, it is not straightforward as to what format such information should be available, i.e. verbal, written information, through, hard copy (booklets/brochures) or soft copy (websites/blogs).
- Benefits could surely derive from the establishment of a common European information portal in paediatric cancer care. It would avoid redundancies and aim at harmonization, while providing supervision of the quality and correctness of information available on the internet. It would provide common standards. The importance of translation into local languages is also clear.
- Language can represent a barrier not only at the level of English/national language, but also at the level of national language/immigrant language: for example in Greece where numerous with Turkish immigrant patients and their families face major communication problems not understanding Greek, or Italy with Albanian/Chinese immigrants who do not understand Italian. It is not clear at this stage how such language and cultural barriers can be tackled
- Some Member States are particularly poor at providing sound, comprehensive information to patients and their families. In particular patients in Bosnia-Herzogovina and Serbia face major problems understanding their illness due to a lack of adequate training among professionals on the importance of providing comprehensive information to patients.
- Countries at the positive extreme of the spectrum include Sweden. The Swedish Childhood Cancer Foundation provides up-to-date and easily understandable information through booklets as well as online resources. In the UK Children Cancer Leukaemia Group (CCLG) provides a range of papers and digital publications to support patients and their families.
- The national expert from the Czech Republic pointed out the need for the establishment of a national parental/patient and survivors organization.

### 4.6 What has been the impact of European funding so far?

- To note, this question seems to be the most difficult to answer. Not many investigators seem to be aware of the effect of European funding on their national paediatric oncology care and research. A number of respondents failed to answer this question, i.e. Germany, Czech Republic, Serbia, Slovakia and Turkey. Does this scarce knowledge imply also that they did not take the best possible advantage out of the European funding? If so, what could be done to disseminate/spread better knowledge about European funds? This should be considered.
- Countries which explicitly complain about the very limited or null EU funding so far are the UK, Belgium, France, Ireland, Malta, Poland and Spain.
- The most cited impact of European funding from the responses is the SIOPEN project, known as the European Neuroblastoma Research Network). Other specific projects cited include the establishment of an European neuroblastoma database (as noted by the report from Denmark), the ‘Europe against Cancer Program’ (noted by the Greek expert), ‘PANCARE’ project, *a European network of professionals, survivors and their families established to ensure that every European survivor of childhood and adolescent cancer receives optimal long-term care* (highlighted by the national reports from Italy and Slovenia), ‘ENCCA—the European Network of Cancer research in Children and Adolescents’ (as outlined by the reports from Poland and Slovenia).

### 4.7 What are future areas that need to be addressed by the European Commission?

- A common problem for small countries, indeed almost all countries, is the characteristic rarity of the diseases. This (the low number of cases per year represents a major obstacle to implementing clinical trials on a national basis. International clinical trials are thus necessary to organize. It was concluded by many experts that increased EU funding to facilitate the running of international clinical trials could play a fundamental role in providing better treatment and care for Europe’s young cancer patients.
- There is an urgent need to facilitate the implementation of clinical trials in Europe: the 2004 EU CTD has made such activities hugely burdensome and extremely expensive. There needs to be an immediate reduction in the administrative and regulatory burden currently affecting the clinical and scientific community working on this rare disease.
- The establishment of an ‘international trial bureau’ for multicentre paediatric academic clinical trials could surely benefit and accelerate paediatric oncology research at the European level: the national expert from Belgium suggests that the EORTC should be a major player in this.
- Support should be put in plave for the establishment of an international parental/survivor organization or strengthening ICCCPO: towards better communication (see # 4).
- It would be useful to support the establishment of a European registry for paediatric oncology for the collection of epidemiological data, as well as the establishment of tissue storage.
- There should be support for the establishment of pan-European guidelines for treatment and greater efforts towards harmonization of treatment across European countries.
- Advanced training should be promoted for medical doctors.
- Collaboration with Mediterranean countries for the establishment of outreach clinical trial programmes could be highly beneficial for such rare diseases.
- A common thread in the responses received include promoting centralization of services and facilities, avoiding fragmentation at all levels and redundancy of the bureaucratic aspects of treating paediatric cancers, particularly by establishing a common platform or infrastructure. For IRB (Institutional Review Board) review, for repository for clinical trials, for guidelines concerning treatment protocols, for support for families and supervised updated information about paediatric cancers, for epidemiological and genetic data, for off-label drug use, etc.

## Austria

**Table:** 

Name	Assoc. Prof. Ruth Ladenstein, M.D., MBA, cPM
Country	Austria
Position	Assoc. Prof. of Paediatrics, Senior Haemato-Oncologist
Institution	St. Anna Children’s Hospital St. Anna Kinderkrebsforschung e.V. Children’s Cancer Research Institute

### 1. How is paediatric care and research delivered in Austria?

There are six major centres in Austria accruing patients in paediatric oncology trials.

- St. Anna Children’s Hospital/paediatric oncology department (which is closely connected with the Children’s Cancer Research Institute (CCRI) in Vienna)
- Medical University of Vienna/paediatric oncology department for brain tumours
- University Hospital Graz/paediatric oncology department
- University Hospital Innsbruck/paediatric oncology department
- Regional Hospital Linz/paediatric oncology department
- Regional Hospital Salzburg/paediatric oncology department

Plus three smaller regional hospital centres in Leoben, Dornbirn and Klagenfurt not providing the full paediatric oncology portfolio but interacting with the larger centres as outlined above.

In Austria research for paediatric oncology is mainly funded through donations. There is no direct financial support from the Austrian government or the City of Vienna, other than grants for competitive, peer-reviewed research projects.

The following funding bodies and programmes have previously supported research in paediatric oncology:

- Fonds zur Förderung der wissenschaftlichen Forschung (FWF)
- Forschungsförderungsgesellschaft (FFG)
- Framework Programmes by the EC
- Gen-AU Program by the Austrian Federal Ministry for Science and Research (BM:WF)
- Jubiläumsfonds der Österreichischen Nationalbank (ÖNB)
- Medizinisch-Wissenschaftlicher Fonds des Bürgermeisters der Bundeshauptstadt Wien
- Wiener Wissenschafts-, Forschungs-und Technologie Fonds (WWTF)

There is no official established network for paediatric oncology in Austria although centres are working in close contact. Within the Children’s Cancer Research Institute in Vienna a unit to run all Austrian paediatric oncology trials has been created (S2IRP Studies and Statistics on Integrated Research). This unit serves as a central quality platform for Austria with international links for data management with GCP-conformity for study design and statistical analysis.

### 2. What are the key issues for paediatric oncology in delivering care and research in Austria?

- Running clinical trials under the EC-directive and its implementation into the Austrian drug law in 2004 with the manifold issues concerning the sponsor role, insurance, monitoring, to highlight a few examples.
- Problems in dealing with drugs in off-label use or non-licensed drugs vis-à-vis the Austrian Competent Authorities and Ethics Committees.
- Lack of a publicly funded network for research and drug development in paediatric oncology. Since 2003 applications have been handed in repeatedly to the Ministries of Health and Science to achieve public funding for the foundation of such a network
- To perform the necessary tasks on behalf of patients
- To allow drug development, to increase visibility of Austria
- To be able to become part in larger international network structures.

### 3. What are the sources of funding for your paediatric oncology research. What are your views on the balance between public and commercial sources of income?

As outlined above, we would like to create an official network in Austria which would allow integrating public funding, industrial support and eventually private donations.

### 4. The state of patient information in Austria for childhood cancers

The CCRI is running a website (http://www.ccri.edu) at which also holds links to other websites. Additional information is provided more specifically related to events and distributed through media. Potentially, Austria could take benefit from a common European information portal in paediatric cancer care, ideally translated into German. Many of our patients will also consult the German website http://www.kinderkrebsinfo.de.

### 5. What effect has European level funding (Framework Programme etc.) had on European paediatric oncology?

For neuroblastoma we had the opportunity to coordinate the SIOPEN-R-NET within the 5th Framework Programme EC grant no. QLRICT-2002-01768.

Science Communication Project DIRECT within the 7th Framework Programme. ENCCA (European Network for Cancer research in Children and Adolescents), within the 7th Framework Programme, currently in the negotiation phase.

We had the opportunity to participate in a number of EC funded projects either as a participant or in the role of project coordination.

### 6. Key areas to be addressed by the Commission in the next 5 years

- Drug development in paediatric oncology
- Funding for paediatric oncology research
- to enhance understanding of the disease spectrum allowing for new insights and hence
- better drug development programmes to improve outcome in these rare diseases to foster controlled clinical trials and registries.

## Belgium

**Table:** 

Name	Benoit Yves
Country	Belgium
Position	Head-Full Professor
Institution	Ghent University Hospital

### 1. How is paediatric care and research delivered in Belgium?

I prefer to use ‘paediatric hemato-oncology’ instead of ‘ped. Oncology’ because we have always mixed experts/units in Belgium. Until recently there were four larger PHO units and four smaller PHO units (eight in total). Of course is large and small relative because the numbers for Belgium are limited (only 10.5 million inhabitants). In Belgium means ‘Larger centres’ more than 50 new patients/year. Smaller is less than 40 new patients/year. There is until now no limitation for other hospitals to take care of children with cancer but these hospitals are not founded to do this care. In reality this is a rare event (maybe only more frequent for brain tumours). A recent proposal on reorganization of the PHO landscape is than we will have two levels in these centres:

1. Principal Treatment PHO Centres (PT PHOC) with more than 50 new patients/year. Because two smaller units will merge, we will have five PTPHOC.
2. Satellite PHO Centres: these centres shall work together closely with a PTPHOC. There will be two left because two small will fuse to one large.

The situation then will be that we will have seven instead of eight centres. This is not yet realized but all preparative work is done and the financial aspects is also agreed on. So it seems to have a great change to be realized. All this new-defined PHO centres work together in scientific programs and clinical trials under the umbrella of a national society: the Belgian Society of Paediatric Haemato-Oncology. The basic and translational PHO research is mostly part of larger research units in Universities or University Hospitals. The clinical leukaemia research is done with the EORTC Children Leukaemia Group. There other clinical trials are conducted with other groups outside Belgium. Until now (except for the leukaemia where EORTC is the sponsor) the national sponsor is always done by a treatment centre that functions as main sponsor for the other Belgian centres. But this could change (see later).

### 2. What are the key issues for paediatric oncology in delivering care and research in Belgium?

As said we have just finishing a fruitful round with the Government in the optimization of the care at the PHO centre level and also in the structuration of the Networking at the national level. It was not possible to go for a better centralization and limiting the number of centres also because we lacked formal documents from leading societies in Europe. The sentence in the National Institute for Clinical Excellence (NICE) document that states so clearly ‘safe and effective services as locally as possible, not local services as safely as possible’ was only partially fulfilled. Indeed the local administrations in hospitals were too strong to overcome this obstacle. So we ended in PT PHOC and Sat PHOC. The program of cancer care will limit the care to these centres. The delineation in age (versus medical oncology and adult haematology) is below the age of 16 years.

This whole PHO cancer process of restructuration is however not finalized. We can send you the document that I wrote (100 pages) but it is in Dutch. We have embedded major psychosocial support to families with a child with cancer, we have also organized the palliative care in liaison equips between PT PHOC and regional hospitals and home care.

We will have also a start of a national platform for clinical trials. We could call this a ‘national trial bureau for childhood cancer’. Of course other countries around us have this structure already. As you will realize Belgium has only 300 new cases a year and this will never allow us to conduct tumour specific clinical trials in our country. So given the new rules in EU (2004) we will need a platform where we can work better in an international context (something that we did of course before the EU directives). We have still a lot of work in Research: Clinical trials and also translational research.

### 3. What are the sources of funding for your paediatric oncology research. What are your views on the balance between public and commercial sources of income?

Governmental Research Founds: within this resources there are different ways.

- National research founds
- University research founds.

Cancer Leagues

- National research founds
- Community (= regional—Belgium has two communities) research funds

Local Childhood Foundations. Some are strong foundations like the Children Cancer Foundation (Ghent).

EORTC: Helps and promotes academic clinical and translational research cancer. We are members of the Children Leukaemia Group. The EORTC is founding partially our clinical research. Europe resources but this is very limited for us.

### 4. The state of patient information in Belgium for childhood cancers

This is mostly given by the treatment centres, by the parents associations from the centres (there are no national and by the national cancer leagues. Because we have two major languages (Dutch and French) the cancer leagues are mainly community oriented.

### 5. What effect has European level funding (Framework Programme etc.) had on European paediatric oncology?

For the moment I only see that some centres have been co-founded by Europe through other initiatives like neuroblastoma, osteosarcoma and Euramos.

### 6. Key areas to be addressed by the Commission in the next 5 years

Facilitate (legislation or directives) academic clinical research for paediatric oncology that has very often an international dimension. The EU directives have induced huge problems for paediatric academic research. Great countries have sometimes the possibility to organize them selves on the national level but that is impossible for small countries. All countries have serious problems in organizing this research from the national level to the international level.

#### Fund of an international trial bureau for paediatric academic clinical trials

As member of the EORTC I have always seen the possibilities for EORTC as structure that could take a role in organization of clinical trials on the European level. EORTC has the knowledge and the expertise to work internationally and also intercontinental (especially with the US and NCI). Of course, I know that previous attempt to talk were not very fruitful and will make possible interactions not easy. Major change in attitudes will be necessary: 1. From EORTC that is mainly an adult oncologic world. EORTC has during the last years changed in many ways and has understood that their role is not in competition with national groups but in organizing the international aspects. They are anyway completely in line with the goal: academic clinical research. EORTC is also very much embarrassed by the EU directives but they try very hard to work according these excessive rules and that makes them in the eyes of ‘one country oriented’ groups a complex organization. But that is the whole challenge for EORTC, to go international. 2. The willingness of the countries to invest in this structure. Europe could help a lot by founding such a structure.

#### Promote and fund translational research

Pharmaceutical aspects: harmonization of the indications and the authorization to prescribe the drugs would be an important point. Patient related aspects: Legislation on the European level for better assurance (live and heath) for cured patients (all patients with chronic diseases).

## Bosnia-Herzegovina

**Table:** 

Name	Jelica Samardzic Predojevic
Country	Bosnia and Herzegovina, Republic of Srpska
Position	Chief of Department Children’s Hemato Oncology
Institution	Children’s Hospital Banja Luka

### 1. How is paediatric care and research delivered in Bosnia and Herzegovina?

Bosnia and Herzegovina has two entities with completed separated system of health care—Republic of Srpska and Federation of Bosnia and Herzegovina.

Republic of Srpska has only one centre for paediatric oncology in Banjaluka. Because of very low budget, there are no research funded in general, especially in paediatric oncology.

### 2. What are the key issues for paediatric oncology in delivering care and research in Bosnia and Herzegovina?

There are no:

- adequate hospital space
- well educated radiologist in paediatric oncology
- low level of nurse’s education in paediatric oncology
- adequate diagnostic procedures—everything is adapted to adult patients.

### 3. What are the sources of funding for your paediatric oncology research. What are your views on the balance between public and commercial sources of income?

Not specified.

### 4. The state of patient information in Bosnia and Herzegovina for childhood cancers

Patients have got information from health-care professionals, some patients information brochures, etc.

### 5. What effect has European level funding (Framework Programme etc.) had on European paediatric oncology?

Not specified.

### 6. Key areas to be addressed by the Commission in the next 5 years

Education of health-care professionals (doctors, nurses, psychologist, social workers).

## Czech Republic

**Table:** 

Name	J. Sterba, J. Stary, T. Eckschlager
Country	Czech Republic
Position	Chairpersons of Czech Paediatric Oncology Working Group (J. Sterba-Chair Elect, T. Eckschlager-Past Chair) and Czech Paediatric Haematology Working Group (J. Stary)
Institution	Department of Paediatric Oncology, University Hospital Brno Department Paediatric Haematology and Oncology, University Hospital Motol, Prague

### 1. How is paediatric care and research delivered in Czech Republic?

Care of children suffering from solid tumours is provided by two centres in Prague and in Brno, and from leukaemia also in six other paediatric departments of large hospitals (České Budějovice, Plzeň, Ústí n Labem, Hradec Králové, Olomouc, Ostrava) Research is funded mainly by grants provided by national and sometimes also by EU grant agencies. Project of childhood cancer registry and tissue bank is funded by Ministry of Health from this year.

### 2. What are the key issues for paediatric oncology in delivering care and research in Czech Republic?

The main problem in clinical care is the increasing burden of the administrative work connected to the opening and realization of the clinical studies which is not reflected by financial and personal support neither by the state (Ministry of Health) nor by the insurance companies and thus nor by hospitals. Many ‘classical’ chemotherapy drugs (actinomycin, merkaptopurin, asparaginase, procarbazine, etc.) lost their registration for clinical use in the Czech Republic and we have to ask the insurance company for the agreement with the treatment of every individual patient. This is time consuming and useless administrative work.

The main problems of research are short-time financial support provided by grant agencies—the usual duration of grants is 3 years only—and lack of adequate support for young physicians/researchers’ education from hospitals or universities to attend nonprofit academy international congresses, hospital stays etc which may help to harmonize the level of knowledge across European states.

### 3. What are the sources of funding for your paediatric oncology research. What are your views on the balance between public and commercial sources of income?

Researches are funded mainly by public (state, university) grant agencies in Czech Republic, part of research are also covered by charity foundations. Commercial sources support mainly drug clinical studies. There were almost no commercial studies for children with cancer since 2002 (backed by pharmaceutical companies) but recently the situation is slowly improving. Administrative and financial burden is also limiting the number of investigator initiated clinical trials. General public opinion here in the Czech Republic is that the main source of research funding should be the state—Ministry of Health, Universities, other grant agencies.

### 4. The state of patient information in Czech Republic for childhood cancers

General informed knowledge on childhood cancer is quite good and is provided by paediatric oncologists, general paediatricians and by charity foundations.

Gap—there is neither national parental nor survivor’s independent organization to date in the Czech Republic. Support for such entity highly appreciated.

### 5. What effect has European level funding (Framework Programme etc.) had on European paediatric oncology?

Not specified.

### 6. Key areas to be addressed by the Commission in the next 5 years

The most important area for next years is help with international non-commercial studies, or investigator initiated studies, including their legal aspects. Enrolment of children into the therapy optimizing trials should be defined as the standard of care in paediatric oncology. EU CTD 2001/20/EC should be modified to enable more realistic daily clinical practice. Commission may also consider supporting pan-European paediatric guidelines (standards of care) creation like PanCare project for survivors—supportive care guidelines, transplant or disease-specific guidelines etc.

## Denmark

**Table:** 

Name	Henrik Schroeder
Country	Denmark
Position	Consultant, Pediatric Oncologist
Institution	Aarhus University Hospital, Skejby

### 1. How is paediatric care and research delivered in Denmark?

In Denmark 150–160 new cases of cancer in children below 15 years of age each year. All children up to the age of 15 years at the time of diagnosis are treated at the four Danish centres:

- National State Hospital Copenhagen: 70 cases
- Aarhus University Hospital, Skejby: 45 cases
- University Hospital Odense: 25 cases
- University Hospital Aalborg: 13 cases

We have a total of about 20 specialists involved in paediatric hematology and oncology daily.

Clinical work is supported by the Danish Regions. The four centres collaborate in using the same international protocols for patients with cancer and we take active part in the international collaboration. We have two paediatric professorships (one in Copenhagen, one in Aarhus) primarily funded by private foundations with minor funding from the universities. We have presently about 16 ongoing PhD studies primarily funded by private foundations.

The Danish Childhood Cancer Foundation distributes about 750.000 Euros every year for paediatric research in Denmark. The Danish Medical Research Foundation and the Danish Cancer Society also contribute with funding for paediatric oncology research. Also the health authorities contribute with funding among other things to the Danish Childhood Cancer Registry.

### 2. What are the key issues for paediatric oncology in delivering care and research in Denmark?

Our goal is to participate to the steady development and improvement of the care of children with cancer by treating all patients according to international protocols and to report clinical data to the various international studies. Most of the research programmes that have Danish patients include all Danish patients with the cancer in question or cover Nordic/Baltic cohorts.

### 3. What are the sources of funding for your paediatric oncology research. What are your views on the balance between public and commercial sources of income?

About 75% of the resources for paediatric research stem from private foundations, primarily the Danish Childhood Cancer Foundation, The Danish Cancer Society and the NOVO Nordic Foundation but also from other smaller private foundations. About 25% comes from public institutions (The Danish Medical Research Council and the Universities). Only a negligible funding comes from commercial instances (the pharmaceutical industry). Our view is that a greater part of paediatric research should be funded by the health authorities.

### 4. The state of patient information in Denmark for childhood cancers

We have written regularly updated information on all the major childhood cancer groups available on the home page of the Danish Childhood Cancer Foundation. A new home page for the Danish Association of Pediatric Hematology and Oncology (DAPHO) supply information on paediatric cancer in Denmark, and also the home page of the Danish Cancer Society provides valuable information.

### 5. What effect has European level funding (Framework Programme etc.) had on European paediatric oncology?

To my knowledge, European Funding has been achieved only for two projects: the SIOPEN collaboration with establishment of a European neuroblastoma database, and the Loulla & Philla liquid formulation of anticancer drugs to children.

### 6. Key areas to be addressed by the Commission in the next 5 years

- Funding of PhD studies involving multinational studies in Europe.
- Funding of research nurses for reporting of clinical data to the various international collaborative studies.
- Funding of travel expenses for national coordinators of international research protocols.

## France

**Table:** 

Name	Francois Doz
Country	France
Position	MD, Paediatric Oncologist and Deputy Director for Teaching and Research
Institution	Institut Curie

### 1. How is paediatric care and research delivered in France?

30 units. Networks:

- National network: SFCE
- International network: SIOPE
- 10 SFCE centres are part of ITCC

### 2. What are the key issues for paediatric oncology in delivering care and research in France?

#### Care

The principal issue is the establishment of a good networking from home to real tertiary care. Also, special needs: radiologists, pathologists, nurses. For research:

- Funding
- Link with true fundamental research
- True implication of industrial companies, sponsoring early drug development studies.

### 3. What are the sources of funding for your paediatric oncology research. What are your views on the balance between public and commercial sources of income?

#### Research funding

- Public grants: France (majority), Europe (minority)
- Private grants (cancer charities, specific paediatric cancer charities)
- Industry: only for a minority of early drug development studies.

Funding of my personal paediatric oncology research in the last years:

- National grants (‘PHRC’) & Charities: RETINOSTOP, ARC, Enfants & Santé
- Institutional (Institut Curie and Assistance publique—Hôpitaux e Paris)
- Industrial grant.

Real industrial support should be possible for early drug development studies. European incentives have not been really efficient to facilitate studies giving access to new drugs.

### 4. The state of patient information in France for childhood cancers

Main gaps are information regarding:

- epidemiology
- clinical research.

### 5. What effect has European level funding (Framework Programme etc.) had on European paediatric oncology?

Effect of European funding:

- Facilitates translational research networks
- Few (but not null) impact on clinical research until now.

### 6. Key areas to be addressed by the Commission in the next 5 years

Key areas the commission could address: one important challenge is to try to develop more and more large international collaboration not only in the field of clinical studies but also in the field of epidemiological, translational and fundamental research. High throughput techniques provide an amount of data on a new scale: it is necessary to increase scientific exchange in order to be able to analyse these new data. The classical competitive approach between research teams must be completed by a more integrated clinico-biological approach: European funding could contribute to encourage and support a new type of scientific collaboration and sharing of data.

Numerous methodological aspects of clinical research are also evolving (new drugs evaluation, use of emerging biomarkers, implementation of novel cellular, tissue and organ imaging studies…): rather than developing them only locally in a non reproducible fashion, European funding could contribute to the establishment of consensus methods which will allow a faster and better comparison of data.

## Germany

**Table:** 

Name	U. Creutzig and T. Klingebiel
Country	Germany
Position	Prof. Dr. Thomas Klingebiel: President of the GPOH Prof. Dr. Ursula Creutzig: Manager GPOH
Institution	Society for Pediatric Oncology and Hematology (GPOH)

### 1. How is paediatric care and research delivered in Germany?

More than 30 cooperative nationwide study groups from Germany and Austria are cooperating in an international setting or integrated in an international network (see table attached). In Germany, these trials and the involved clinic centres cover >90% of the incident patients. All patients are registered in the German Childhood Cancer Registry (GCCR) http://www.kinderkrebsregister.de/english/

Research is often connected with these studies. Basic research includes molecular genetics, immunology, pharmacogenetics, minimal residual disease for the different leukaemias and various tumours. There are around 30 big units/institutions/clinics performing research and more than 54 clinics involved in the cooperating studies (see: http://www.kinderkrebsinfo.de/e67296/e4863/index_ger.html

Clinical and basic Research in paediatric oncology is mainly public funded by the German Krebshilfe (http://www.krebshilfe.de) and the Deutsche Kinderkrebsstiftung (http://www.kinderkrebsstiftung.de). Additionally the study infrastructure is supported sometimes by local parent organizations. The German Research Foundation (Deutsche Forschungsgemeinschaft, DFG) http://www.dfg.de/ and the German Ministry of Education and Research (BMBF) is funding mainly basic and translational research. Different study groups take part in ENCCA, a European funded Network of Excellence for Cancer in Children and Adolescents. There are several foundations which support also paediatric oncology projects. However, in comparison to other European countries (e.g. UK), infrastructure funding for clinical trials in general, including paediatric trials, is rudimentary. Funding for individual studies needs to be applied from various sources. In addition, for each trial funding for infrastructure both at the level of the study centre and at the level of the participating institutions has to be applied. This lack of a funded trial infrastructure makes funding of individual studies extremely expensive for charities as well as other funding bodies.

### 2. What are the key issues for paediatric oncology in delivering care and research in Germany?

The GPOH’s purpose is:

- to provide the highest cure rates with less late effects for children with cancer nationwide.
- to investigate the pathology, diagnostics and treatment of haematological diseases and cancer in children and adolescents and to optimize the corresponding structural conditions.

The society promotes training and advanced education for all professional groups in paediatric haematology and oncology. The society aims at collaborating with other national and international societies for paediatric haematology and oncology, as well as with the Society for Paediatrics and other associated societies, formations and self-help groups.

### 3. What are the sources of funding for your paediatric oncology research. What are your views on the balance between public and commercial sources of income?

The main sources of funding for our paediatric oncology research are described above (point 1). Commercial sources of income concern clinical trials of phase I and II are low. The financial support needed for the nationwide cooperating clinical trials is >10 million Euros per year—however, provided is much less. Projects funded by the DFG and the BMBF (national level, federal resources) are in the vast majority of cases not related to clinical trials and registries.

### 4. The state of patient information in Germany for childhood cancers

Patient information is given for each trial in detail (for parents, patients in different age groups). It is also available for patients and parents for most diseases on http://www.kinderkrebsinfo.de/e1812/index_eng.html and is distributed in printed versions by the Deutsche Kinderkrebsstiftung. We are trying to reduce the gaps.

### 5. What effect has European level funding (Framework Programme etc.) had on European paediatric oncology?

Not specified.

### 6. Key areas to be addressed by the Commission in the next 5 years

- Reduce administrative burden (i.e. the regulatory requirements) for academically driven clinical trials.
- To build up a ‘one stop shop’ for contact and exchange between academia and industry. There is indeed an urgent need for a platform through which multinational clinical trials may be harmonized and funded independent of industry support (‘orphan situation’ of Paediatric Oncology).
- Achieve a stable long-term funding for clinical trials.
- Harmonization of tissue storage techniques and improve biological studies with epidemiological data.
- Improve the clinical MD/PhD training programmes for young professionals.
- Improve the recognition of Paediatrics for future initiatives or collaboration at the European level since it has specific needs that are not necessarily found in adult cancers.

## Greece

**Table:** 

Name	Helen V. Kosmidis
Country	Greece
Position	Director of Paediatric Oncology Department
Institution	Children’s Hospital ‘Aglaia Kyriajou’ Athens

### 1. How is paediatric care and research delivered in Greece?

In Greece, there are six paediatric oncology departments for approximately 280–300 new patients per year. Of them three are located in Athens, two in Thessaloniki (northern Greece), one in Crete. In addition one SCT unit is available in Greece for children only. Of these six units, three belong to the University and three (in which the majority of children are admitted) belong to the National Health system. Also of these six, two are purely Oncology departments and four are paediatric Hematology-Oncology departments. Oncology departments in general follow European protocols and participate in some of them such as hepatoblastoma, HR neuroblastoma, Wilms’ tumour, BFM ALLIC etc.

Paediatric Oncologists and paediatric Hematologists-Oncologists are members of the Hellenic Society of Paediatric Hematology-Oncology. Also, paediatric hematology-oncology as subspecialization has been proposed by our Society and accepted by the Ministry of Health: According to the proposal, candidates should be paediatricians (4 years of general paediatrics) and then complete 3 years in paediatric hematology-oncology in organized centres. Among 3011 paediatricians, 78 are specialized paediatric Hematologists–Oncologists and of these 41 are working in the departments described above.

Research is mainly funded by the University and the Central Advisory Health Committee of the Ministry of Health. Some projects are covered by the annual money raising activity of the Hellenic Anticancer Society again through the Central Advisory Health Committee and few by the ‘Europe against Cancer program’ of EU.

Only recently, paediatric cancer registry is available in Greece. Supporting groups in Greece are parent groups (Floga and Pisti in Athens, Lampsi in Thessaloniki, Iaso in Larisa, Artemis for Histiocytosis). Among the parent groups, Floga and Iaso are members of the International Parent Group Organization (ICCPO). Except for parent groups, other supporting groups are those of ‘friends’ of children with cancer such as Elpida, Storgi, Iliachtida etc. Recently a survivor group was founded and was named ‘kyttaro’ (cell).

### 2. What are the key issues for paediatric oncology in delivering care and research at national level?

Optimal care is provided in our paediatric oncology departments although there is nurse shortage. Medical and supportive care are both of high standards. In our Hospital, a new paediatric radiotherapy department fully equipped is to start next October for children and adolescents. However research at national level is left behind (with exceptions as is the collaboration of all six Oncology departments with the Department of Hygiene and Epidemiology of the University of Athens) and our scientific activities are rather restricted to clinical level.

### 3. Please describe in greater detail the sources of funding for your paediatric oncology research. What are your views on the balance between public and commercial sources of income?

As described above. In addition there is no commercial source of income.

### 4. Please describe the state of patient information in your country for childhood cancers, including who provides this information. Are there any gaps in your view?

Patient information is provided by the physician in charge and is based on the age, maturity and culture. Communication is verbal usually with the presence of parent(s), psychologist and/or nurse. Specially edited pamphlets are also given to parents and patients. One of the problems faced is related to language barrier between the health-care team and the economically immigrant families.

### 5. What effect has European level funding (Framework Programme etc.) had on European paediatric oncology?

Not specified.

### 6. Key areas to be addressed by the Commission in the next 5 years

It will be greatly appreciated if countries as ours could be incorporated and funded in various projects. As an example we had welcome the invitation by FECS special project (collaboration between nurses and doctors) to participate.

## Hungary

**Table:** 

Name	Assoc. Prof. Miklós Garami, MD., MSc., PhD.
Country	Hungary
Position	Chief, Unit of Oncology
Institution	2nd Department of Paediatrics, Semmelweis University

### 1. How is paediatric care and research delivered in Hungary?

The history of paediatric oncology in Hungary began with the foundation of the Hungarian Paediatric Oncology Network in 1971. The activities of our group consisted of: (1) registration and follow-up of the patients, (2) treatment by the same protocols nationwide, (3) quality control, and (4) postgraduate teaching sessions. It proved to be an important step forward, when the Network received a $2.33 million grant from the US Agency for International Development (USAID) between 1991and 1996. In 2004, an Internet-Based Paediatric Cancer Registration and Communication System for the Hungarian Paediatric Oncology Network was established. In Hungary, health insurance (the compulsory health insurance based on the principle of solidarity) bears the expenses of cytostatic drugs only for children registered and treated according to guidelines of the Hungarian Paediatric Oncology Network. This centralization of the patients’ treatments and regular (annual) audits make oncology centres’ treatment more economical. Assurance of high-quality and economic treatments has been possible by the centralization of all paediatric cancer patients into paediatric oncology centres, the well-organized and reliable paediatric tumour registry and strong international scientific connection of the Hungarian paediatric oncologists.

The Hungarian Scientific Research Fund (Hungarian abbreviation: OTKA) has been the major funding agency of basic science and scholarship since 1986 when the transition to competitive research funding started in Hungary. Its ‘founding fathers’ modelled the principles of operation on the practice of German (Deutsche Forschungsgemeinschaft) and American research funds (National Science Foundation, National Institutes of Health). Upon a government decree, OTKA has been operating as an independent non-profit organization since 1991. Its legal status and rules of operation were established in an act in 1993 and reinforced in 1997 by the Hungarian parliament in order to provide independent support to scientific research activities and infrastructure, to promote scientific achievements of international standards, and to provide assistance to young researchers. As an independent institution, OTKA reports to the parliament and the government of Hungary. With regards to the funds provided within the annual budget of the Republic of Hungary, the appropriations of OTKA are administered via the budget of the Hungarian Academy of Sciences. The administrative and financial tasks related to its operation are performed by the OTKA Office in Budapest.

### 2. What are the key issues for paediatric oncology in delivering care and research in Hungary?

In Hungary, as in most small countries, no institution provides provision for all the complex and expensive diagnostic and therapeutic modalities needed. However, this problem could be solved by a well-organized collaboration with other institutions, significantly reducing the timeframe between the first clinical sign and the diagnosis. The Hungarian Paediatric Oncology Network was organized taking into consideration the guidelines for ‘Paediatric Cancer Centres’ published by the American Academy of Pediatrics (AAP) in 1986, 1997 and 2004 with the help of USAID. The following personnel, facilities and capabilities have major benefits of the quality treatments: (1) Personnel: 24 hours immediate access with primary care physician and/or paediatric oncologist; (2) Facilities: an immediate (within 2 hours) access to a fully staffed, paediatric oncology and/or intensive care unit; (3) Capabilities: blood bank capable of providing a full range of products (including irradiated, cytomegalovirus-negative, and leucodepleted blood components) and access to stem cell transplantation services.

During the two decades of its operation, OTKA has supported approximately fifteen thousand research projects with an overall funding worth 218 million Euros. OTKA’s annual budget for 2008, established in the national currency, is worth about 20 million Euros. It covers the annual financial support of around two thousand research projects (2–4 years of duration each), with 300–400 new research projects starting every year. OTKA administers two rounds of open calls for proposals with a bottom-up approach towards research proposals, postdoctoral research proposals, and proposals for international cooperation every year (often based on bilateral agreements between OTKA and foreign research funding agencies with the view of Europe and Hungary investing in a knowledge-based society)—without thematic restrictions and with a special emphasis on the careers of talented young researchers and on the reintegration of Hungarian researchers returning from postdoctoral trainings or research projects carried out abroad (thus assisting the next generation of researchers and promoting the cooperation between Hungarian and foreign research centres). In addition, OTKA also administers calls for proposals for the establishment of scientific schools directed by internationally acknowledged scientists and for the development of libraries to provide research universities with the opportunity to purchase databases and full-text journals available on electronic media and various networks. A characteristic feature of the calls for proposals is the preference of basic research, involving theory and practice with the primary objective of recognizing new scientific laws and elaborating new methods and skills. (As opposed to the funding policies of the corporate sector investing in targeted applied research and focusing on expected direct results, OTKA does not require specific application and immediate economic utilization since the results of basic research normally occur in the long run.) Competition is very intense: in recent years, the annual figure of applications has varied between 1500 and 1800, the average support ratio being approximately 20% of the total number of proposals received. Their evaluation is based on a peer review system where the individual expert reviews are collected through an online electronic proposal review system and the proposals are further evaluated and ranked by a two-level system of review panels and boards.

### 3. What are the sources of funding for your paediatric oncology research. What are your views on the balance between public and commercial sources of income?

Basic and applied research, development, and innovation rely on two cooperating funding agencies in Hungary: OTKA and the National Office for Research and Technology. However, all research universities, institutions of higher education, and the research institutes of the Hungarian Academy of Sciences primarily depend on OTKA in terms of financing their basic research activities. Complementing its national focus, OTKA has also been an active member of the European Science Foundation since 1996 and the European Heads of Research Councils from 2003, participating in and contributing to their multilateral programmes such as the European Collaborative Research and the European Research Area.

The ratio between public and commercial sources of income is circa. 80% : 20% in the field of paediatric oncology.

### 4. The state of patient information in Hungary for childhood cancers

Primary diagnosis about childhood cancers is provided by an oncology team:

- Paediatric oncologist (M.D.)
- Nurse specialized for childhood cancers
- Psycho-oncologist specialized for childhood cancers.

Verbal, written and electronic information are given for the patients and their family members. These information are offered to help parents and loved ones of a child with cancer know about and cope with some of the problems that come up just after the child is diagnosed. Starting with its effect on parents, then moving on to the child with cancer and the other children in the family (including emotional responses to the cancer and share some ideas for coping).

### 5. What effect has European level funding (Framework Programme etc.) had on European paediatric oncology?

Not specified.

### 6. Key areas to be addressed by the Commission in the next 5 years

The following European level of funding has effect on paediatric oncology (in the order of importance):

- Get to the bottom of ‘off label’ problems
- Support for collaboration in international studies
- EU funding for prevention, screening and early diagnosis
- Financial aid for psychosocial rehabilitation
- Family aid
- Financial support to employ non medical members of the paediatric oncology team
- Support to establish and/or develop internet-based tumour registry for childhood cancer patients.

## Ireland

**Table:** 

Name	A. O’Meara
Country	Ireland
Position	
Institution	Lady’s Children Hospital, Crumlin

### 1. How is paediatric care and research delivered in Ireland?

The National Paediatric Haematology and Oncology Centre (NPHOC) is situated at Our Lady’s Children’s Hospital Crumlin (OLCHC), Dublin to which all newly diagnosed patients with malignant disease in the state are referred. It is also the National Paediatric HSCT centre where autologous and allogeneic, related and unrelated, transplants are performed for indications including leukaemia, bone marrow failure syndromes, haemoglobinopathies, inborn errors of metabolism and certain immunodeficiencies. The NPHOC is supported by 16 paediatric units in local hospitals across Ireland. These paediatric units, termed shared care centres, deliver components of treatment locally in accordance with care protocols agreed with NPHOC. All tertiary paediatric oncology is undertaken in OLCHC, with the exception of neurosurgery, orthopaedic surgery, retinoblastoma-related surgery and radiotherapy. NPHOC is a member of the All Ireland Cooperative Oncology Research Group (ICORG), through which funding from the Health Research Board (HRB) is allocated for the coordination of clinical trials in Ireland. The HRB is a government agency to promote health research in Ireland. The majority of paediatric clinical trials open in NPHOC were coordinated by the former CCLG. More recently, we have become members of the Children’s Oncology Group (COG) and intend to participate in a select group of clinical trials. The National Children’s Research Centre (NCRC) is situated on the campus of OLCHC and is affiliated to the three medical schools in Dublin—University College, Trinity College and the Royal College of Surgeons of Ireland. Basic research, specifically in the area of acute leukaemia, bone marrow failure disorders and neuroblastoma is undertaken with funding provided both by the universities and NCRC following international peer review.

### 2. What are the key issues for paediatric oncology in delivering care and research in Ireland?

The service would be greatly enhanced by having all disciplines, particularly neurosurgery which is currently divided on two sites, based on the age of the patient, at OLCHC. A single tertiary paediatric hospital for the state is planned for Dublin with an expectation that this will be operational in 2014. It is envisaged that all tertiary paediatric oncology services, with the exception of radiotherapy and oncology-related orthopaedic surgery, will be delivered on this site.

From a clinical research perspective, the main issues include:

- The need for separate approval from the Irish Medicines Board for clinical trials which are already approved within the former CCLG
- Provision of ongoing GCP training and education
- SAE collection across the shared care network
- Need to improve the status of electronic transmission of data in relation to clinical trials.

### 3. What are the sources of funding for your paediatric oncology research. What are your views on the balance between public and commercial sources of income?

See above.

Funding is obtained for a small number of clinical trials through CCLG and EU (Hodgkin’s lymphoma trial). The HRB, through ICORG, provides funding for clinical trials; funding is allocated on a points basis depending on the complexity of the trial, following Irish Medicines Board (IMB) approval.

From a basic research perspective, once projects have been internationally approved, the source of funding becomes less relevant.

### 4. The state of patient information in Ireland for childhood cancers

The Children’s Cancer & Leukaemia Group (CCLG) provide information in booklet form on paediatric cancers, transplants etc. In addition, the Irish Cancer Society, the national cancer charity, provides funding and support to produce the patient passport—a parent/patient handbook detailing symptom management at home, hickman care etc. The Irish Cancer Society is also financially supporting the development of a patients’ information website. This project is a work in progress. An information booklet for teenagers is also produced by CanTeen, a teenage support group funded by the Irish Cancer Society. Patient information sheets are not provided for all clinical trials and many patients are treated based on guidelines, none of which have patient information. Information on studies and guidelines is often not provided.

### 5. What effect has European level funding (Framework Programme etc.) had on European paediatric oncology?

Not specified.

### 6. Key areas to be addressed by the Commission in the next 5 years

- Establishing a centralized European IRB for clinical research protocols.
- Survivorship: The provision of a website that includes comprehensive guidelines relating to long-term follow-up of childhood cancer survivors, including access to a software database for survivors that individual European centres can utilize in a certain standardized format, and maintain, thereby facilitating transfer of patients to adult-based follow-up as well as for longitudinal research purposes. This may all be possible through the newly formed PanCare.

## Italy

**Table:** 

Name	Dr Giorgio Dini
Country	Italy
Position	Director, Department of Paediatric Haematology and Oncology
Institution	Istituto G. Gaslini

### 1. How is paediatric care and research delivered in Italy?

In Italy, there are almost 50 centres of paediatric hematology and oncology, which are spread out in the entire country. They are all part of the Italian Association of Pediatric Hematology and Oncology (AIEOP), which is the institution responsible for the accreditation of centres through a systematic quality control. All centres are involved both in research and in patient care within a national public health system, which makes them part of a network.(last question on answer #3)

### 2. What are the key issues for paediatric oncology in delivering care and research in Italy?

The key issues are the Insurance for international studies: it requires for international studies dealing with children an insurance covering at least for 10 years. We are currently struggling in order to have the social security’s cover on this. Public resources to cover research at national level are decreasing with the time.

### 3. What are the sources of funding for your paediatric oncology research. What are your views on the balance between public and commercial sources of income?

About 50% of resources come from public funding. The other 50% comes from private funding, from which 30% comes from non-profit associations and the other 20% from commercial sources. In my opinion, public resources are decreasing, and we are increasingly involved in finding alternative sources.

### 4. The state of patient information in Italy for childhood cancers

During the last 20 years, the quality of the information given to our patients, including children and their families, has significantly increased. All studies are approved by IRB and after an interview with the physician responsible for the treatment, patients over 18 years old, all parents and legal tutors sign an informed consent. Patients under 18 years old are informed about their therapy’s program but do not sign any informed consent. I believe the Italian way of providing patients the information about their treatment and condition works very well and I do not see any gaps on this matter.

### 5. What effect has European level funding (Framework Programme etc.) had on European paediatric oncology?

We have received support for late effect program, within the PANCARE project.

### 6. Key areas to be addressed by the Commission in the next 5 years

We suggest as key area to support an outreach program to increase results obtained in Easter European Countries and Mediterranean Countries in children with malignancies.

## Italy

**Table:** 

Name	Riccardo Riccardi
Country	Italy
Position	Head of the Division of Pediatric Oncology
Institution	Catholic University of the Sacred Heart, Rome

### 1. How is paediatric care and research delivered in Italy?

Medical care is available for all the Italian citizens through the national system that provides standard care free of charge including more complicated procedures like allogeneic bone marrow transplantation. New agents under evaluation are not provided to patients until AIFA (Italian Agency for Drugs) Regulatory Body includes them in the national drugs handbook. No specific plan has been adopted by the government to create a network of paediatric oncology units in respect of the territory and available resources. However, all the Italian paediatric oncology centres from universities and hospitals are part of AIEOP (Italian Association for Pediatric Hematology and Oncology) and the inclusion within this group is given following the evaluation of the requesting centre’s characteristics (attachment 1: self evaluation form). The present number of centres is 54 but this number is above what could be the optimal situation since it includes centres that see less than ten new patients per year. Almost 1 year ago, however, a survey was started to give accreditation only to centres with wide experience and availability of subspecialties like paediatric intensive care unit, radiotherapy unit etc. This goal however is difficult and will be achieved only if there will be a government intervention.

Government fundings for research are very limited. Few centres are involved in research and most of the studies are supported by local foundations. Some activities connected to research as tumour banking at diagnosis are supported by private funds. For specific paediatric oncology questions (minimal residual disease in leukaemia and Myc amplification in neuroblastoma) there are reference centres that are supported by private funds. There are many centres that don’t have a paediatric oncology research lab but they are simply involved in data collection.

### 2. What are the key issues for paediatric oncology in delivering care and research in Italy?

As far as medical care, the excessive fragmentation of patients due to the high number of paediatric oncology centres is explained in the reply to question no. 1.

As far as research is concerned a coordination of research lab and clinics is usually missing in most centres. There are few exceptions related to the major paediatric oncology Institutions in Italy but no more than seven or eight institutions. Centralization at diagnosis of tumour material within centres involved in the treatment of brain tumours is currently an ongoing process, mainly supported by private initiatives such as that by parents organizations or other charity institutions devoted to paediatric tumours.

### 3. What are the sources of funding for your paediatric oncology research. What are your views on the balance between public and commercial sources of income?

The amount of public resources available for paediatric oncology research is quite limited and a consistent amount of funds can be obtained through private organizations even if the mechanism maybe improved. As far as my activity as researcher, this is mainly supported by a Foundation established 20 years ago by Banca D’Italia. In addition another source of financing comes from incomes tax since the amount of 5‰ of taxes can be devoted to accredited research institutes.

### 4. The state of patient information in Italy for childhood cancers

General information about childhood cancer is quite limited and only families in which a paediatric tumour is present will have adequate information. Information are given by clinical staff of the centre where the diagnosis is performed.

### 5. What effect has European level funding (Framework Programme etc.) had on European paediatric oncology?

It’s very difficult to answer this question even though the major paediatric oncology centres in Italy that obtained funding are no more than 5 or 6. However I don’t have data concerning the amount of money obtained through European framework programme by institutes performing basic research.

### 6. Key areas to be addressed by the Commission in the next 5 years

As far as Italy is concerned translational research maybe a key issue (evaluation of prognostic molecular markers and possible target for new agents. This should be part of research programme). Probably approximately ten Italian centres could be able to carry out these studies and could apply for this type of financial support. The creation of fellowships for young paediatric oncologists oriented to lab-clinical research should be supported. All the paediatric patients will benefit from such a possibility. In addition the possibility to continue in Italy the work that has been done abroad should be supported by fellowships or job availabilities when coming back home. Lithuania

## Lithuania

**Table:** 

Name	Jelena Rascon
Country	Lithuania
Position	Senior Physician in BMT unit
Institution	Vilnius University Children’s Hospital

### 1. How is paediatric care and research delivered in Lithuania?

There are two units of paediatric oncology and hematology one in Vilnius university children’s hospital. Children with leukaemia, and the most types (except CNS and endocrinal) of solid tumours are treated in this centre. Paediatric BMT is also performed in Vilnius. The second centre is In Kaunas university where children with CNS, endocrinal and other solid tumours are treated. The research is funded from national sources allocated for research activities as well as from common European projects.

### 2. What are the key issues for paediatric oncology in delivering care and research in Lithuania?

The efforts are concentrated to ensure the quality and appropriate moment to provide the care.

### 3. What are the sources of funding for your paediatric oncology research. What are your views on the balance between public and commercial sources of income?

At the moment commercial source is a minority in paediatric oncology. It plays much more substantial role in adult hematology and oncology research activities.

### 4. The state of patient information in Lithuania for childhood cancers

The information is usually provided by clinicians and psychologist who is working in the department. Many parents gain information from internet.

### 5. What effect has European level funding (Framework Programme etc.) had on European paediatric oncology?

Not specified.

### 6. Key areas to be addressed by the Commission in the next 5 years

To support research in paediatric oncology, to improve the cooperation between the countries in order to enrol more patients, especially from Eastern European countries. Very often centres have no information about clinical trials and research activities in the international level.

## Lithuania

**Table:** 

Name	Lina Rageliene
Country	Lithuania
Position	Ass. Prof., Head of Centre for Pediatric Oncology and Hematology
Institution	Vilnius University Children’s Hospital

### 1. How is paediatric care and research delivered in Lithuania?

The centre for paediatric oncology and hematology in Vilnius university children’s hospital is the main institution for treatment and investigation of paediatric patients with cancer in Lithuania. The small unit for treatment of CNS tumours is located in Kaunas at Kaunas medical university clinics. The care funding is sufficient but research funding is insufficient. The funds are received from national governmental sources and various international grants.

### 2. What are the key issues for paediatric oncology in delivering care and research in Lithuania?

The care funding is tolerable, but research funding must be improved using the international sources of foundation.

### 3. What are the sources of funding for your paediatric oncology research. What are your views on the balance between public and commercial sources of income?

60% of research funding is acquired from Vilnius university and Lithuanian academy of science foundations. 30% we receive from international grants and pharmaceutical companies.

### 4. The state of patient information in Lithuania for childhood cancers

Patients information is provided by all medical personal of our centre. The special informative literature for patients and parents is provided also. The information according different oncological and haematological diseases is presented in websites of three parent organizations. Prior chemotherapy and other treatment of the patients and parents are informed and written consent is received. Gaps are not noticeable.

### 5. What effect has European level funding (Framework Programme etc.) had on European paediatric oncology?

Not specified.

### 6. Key areas to be addressed by the Commission in the next 5 years

The commission should support more the research programs in paediatric oncology. The special interest must be investments and efforts to improve cytogenetic investigations level in all European countries and provide an information and possibility to participate in clinical trials to all European counties.

## Malta

**Table:** 

Name	Dr Victor Calvagna
Country	Malta
Position	Consultant Paediatric Oncologist
Institution	Mater Dei Hospital, Msida, Malta

### 1. How is paediatric care and research delivered in Malta?

Paediatric care in Malta is coordinated by the department of paediatrics at Mater Dei Hospital. The medical director is Prof Simon Attard Montalto and he is answerable to the Director of Institutional Health of the Department of Health in Valletta. The Paediatric Department at Mater Dei Hospital is made up to two general paediatric wards that have 38 inpatient beds. There is also a 12-bedded day care unit on the same floor as the wards. The paediatric oncology ward is also on the same floor and has six inpatient beds. Sick newborns and children under the age of 3 who needs intensive care are admitted to the Neonatal and Paediatric Intensive Care Unit (NPICU). Above the age of 3, sick children are admitted to the adult intensive treatment unit. Outpatients are seen at the children’s outpatients department within the same hospital. This is situated in the main outpatients’ block of the hospital. Emergency referrals are seen at Accident and Emergency Department where there is an area manned by a junior paediatrician who sees all the medical paediatric referrals. Paediatric Surgery is under the headship of the Director of Surgery. There are two paediatric surgeons and the patients are admitted to a 24 bedded paediatric surgical ward. Paediatric research is mainly coordinated by Prof Attard Montalto and funds are provided by pharmaceutical companies and/or the University of Malta.

### 2. What are the key issues for paediatric oncology in delivering care and research in Malta?

Delivering paediatric oncology care locally is somewhat limited because of our small population size and therefore not all the oncology care can be delivered locally. Although most of the patients receive their conventional chemotherapy in Malta, other patients requiring stem cell transplants, high-dose chemotherapy with stem cell rescue, complicated abdominal surgery, e.g. neuroblastoma, specialized radiotherapy, e.g. stereotactic radiotherapy, will have to travel to specialized centres abroad for this kind of treatment. We have direct links with the Royal Marsden Hospital in London and most of the patients go there for specialized care. The oncology ward was not designed to be a paediatric oncology ward although it is a significant improvement on the facilities we had in the old St Luke’s Hospital. The day patients are seen on the same ward as the inpatients. A new paediatric oncology ward is planned for 2013 as part on a brand new oncology block on the grounds of Mater Dei. This will have ten inpatients beds and a four-bedded day care facility. At the moment we treat all patients up to 16 years of age and it is envisaged that with the new ward we will be able to treat children and adolescents up to the age of 18 years. It is very difficult to conduct research in paediatric oncology because of patient numbers and funding. However, if a research fellow is assigned to my unit there is scope for entering patients into international trials and to conduct epidemiological studies on the local scene vis a vis paediatric oncology.

### 3. What are the sources of funding for your paediatric oncology research. What are your views on the balance between public and commercial sources of income?

There are no funds directed towards paediatric oncology research in Malta, at least none that I am aware of. The best way forward is to sponsor a research fellow to our unit with the specific aim of carrying out research in paediatric oncology. This person should ideally be sponsored by the University of Malta but I have no major objections if commercial entities offer their support. Obviously the latter sponsor must not have any vested interest in the project except that of advancing paediatric oncology in Malta.

### 4. The state of patient information in Malta for childhood cancers

All cancers in Malta are statutorily obliged to be reported to the Department of Health Information and this department collates all the data and publishes a yearly report. However, there is no separate report on the local incidence and epidemiology of paediatric cancer and in my opinion this should change if we want to make headway with proper information on paediatric cancer.

### 5. What effect has European level funding (Framework Programme etc.) had on European paediatric oncology?

The new oncology block I mentioned above (for 2103) is being funded partly by the European Framework Programme and this will definitely help to improve paediatric oncology care on the island.

### 6. Key areas to be addressed by the Commission in the next 5 years

Another area that the commission can address is to push for more local research in paediatric oncology by, for example, helping with advice on the type of research and its setup. It can also push for more meaningful health information on the epidemiology of childhood cancer in Malta.

## Netherlands

**Table:** 

Name	Rob Pieters
Country	Netherlands
Position	Chair Advisory Board DCOG
Institution	DCOG

### 1. How is paediatric care and research delivered in the Netherlands?

Seven centres (five oncology, two alloBMT)

### 2. What are the key issues for paediatric oncology in delivering care and research in the Netherlands?

Not specified.

### 3. What are the sources of funding for your paediatric oncology research. What are your views on the balance between public and commercial sources of income?

Childhood cancer research foundation 50%; Dutch cancer society 20%; Others 30%

### 4. The state of patient information in the Netherlands for childhood cancers

Paediatric oncologists and nurses provide information. Gaps: electronic information is insufficient.

### 5. What effect has European level funding (Framework Programme etc.) had on European paediatric oncology?

Not specified.

### 6. Key areas to be addressed by the Commission in the next 5 years

Main support should go to Organization of International collaborative trials in paediatric oncology. The gap between national organizations should be filled. SIOP Europe should be the international body in which all different platforms that now organize International trials (EuroEwing, Interfant, EsPhALL, SIOP-Wilms etc) can be incorporated. SIOP Europe Trial organization should included the chairs of the national organizations (not more than one person per country!!) in their board. SIOP Europe should be the organization that is funded by European money. This should not go to national organizations or networks.

## Poland

**Table:** 

Name	Piotr Czauderna
Country	Poland
Position	Head of the Department of Surgery and Urology for Children and Adolescents
Institution	Medical University of Gdansk

### 1. How is paediatric care and research delivered in Poland?

There are ten medical universities in Poland and each of them has paediatric oncology departments. Apart from this there are two research institutes located in Warsaw (the capital): Children’s Memorial Hospital and Institute for Mother and Child. Both of them have paediatric oncology departments, too, although in the latter case it is combined with paediatric oncology surgery unit.

All the departments form the national network within two groups: Polish Group of Leukemias and Lymphomas and Polish Pediatric Group for Solid Tumors. Each of the groups has different working-research tumour groups which are run by national coordinators and their native centres. This is largely based on international cooperation, i.e. Polish Wilms’ Tumor Group cooperating with SIOP, Polish Soft Tissue Sarcoma Group cooperating with CWS, Polish Liver Tumors Group cooperating with SIOPEL, Polish Neuroblastoma Group cooperating with SIOPEN, Polish Rare Tumors Group, Polish Osteosarcoma Group, Polish Ewing Group cooperating with EuroEwing and Polish Germ Cell Tumors Group. Apart from this, the Polish Society for Pediatric Hematology and Oncology exists based upon individual memberships.

Research is funded generally in two ways: directly by the medical school and institutes (which allows for small money only—around 7000–10,000,—Euros per institution, so called statute purposes research) or through national grants funded by the Ministry of Science and Education. Twice a year applications are collected followed by independent review and funding selection. This may be up to 25,000–100,000 Euros for the period of 3 years maximally. There is also some charity money involved from parent organizations but this is of rather small significance for research funding.

### 2. What are the key issues for paediatric oncology in delivering care and research in Poland?

The main problem have been legislative and administrative issues resulting from the EU CTD implementation. As a result of them no single international trial has been opened in Poland since 2007. This is mainly caused by the reluctance of academic institutions taken the role of national or international sponsors and by the costs resulting from opening of new trials: patients’ insurance and administrative costs which are between 3,000 and 5,000,—Euros per trial which can’t be funded through the grant system. Recently there has been some progress in this area with possibility of contracts being signed between internationally recognized sponsors and polish coordinating institutions responsible for selected tumours coordination in nearby future.

### 3. What are the sources of funding for your paediatric oncology research. What are your views on the balance between public and commercial sources of income?

I have written about it in the point above. There is no balance between public and commercial sources of income for research funding, whatsoever. Very little commercial sources of funding are included in paediatric oncology trials. Pharmaceutical industry has little interest in running paediatric oncology trials and what is even worse this sort of decisions are taken not at the national level of company’s management but at their international headquarters, so the chance for commercial support are minimal.

There is also a possibility to apply for the European grants within FP7 program but the chance for success is very small.

### 4. The state of patient information in Poland for childhood cancers

In my opinion state of patients/parents information in Poland for childhood cancers is still insufficient as it comes mainly from informal sources, i.e. informal web-based parents discussion lists or personal internet search, or parent-to-parent communication. There are some parental organizations (NGOs) but they are still relatively week in my opinion. Probably there is only one relatively strong survivors organization which support mainly a single centre only. There are, however, some charity foundations which support children with cancer and their families, i.e. in fulfilling their dreams. Nevertheless I don’t see an organized and well-concerted action providing objective information to parents and patients taking the national picture as a whole. Of course children who participate in international/national trials are adequately informed through proper information sheets and consent forms which need to be nowadays approved by the local ethical bodies in every case.

### 5. What effect has European level funding (Framework Programme etc.) had on European paediatric oncology?

In my opinion there has been little effect of the European level funding (Framework Programme etc.) on European paediatric oncology until now, although the recent ENCCA network of excellence grant awarded may change this picture a bit.

### 6. Key areas to be addressed by the Commission in the next 5 years

- Researchers-driven (Academia sponsored) paediatric clinical trials, particularly those focused on rare tumours. However one has to keep in mind that rare tumours definition in the world of adult oncology has very little to do with paediatric cancers. All our tumours are then super-rare!
- Facilitation of the paediatric clinical trials by creating a single application (English) file which would not have to be translated to other languages which bears unnecessary costs. It would be even better, if it might be submitted to a single pan-European body in order to save the work on national level.
- Trial calls dedicated specifically for children promoting global overseas cooperation and introduction of translational research into clinics.

## Poland

**Table:** 

Name	Jerzy R. Kowalczyk
Country	Poland
Position	Professor
Institution	Department of Paediatric Haematology, Oncology and Transplantology, Children’s University Hospital, Lublin

### 1. How is paediatric care and research delivered in Poland?

Polish population aged 0–18 years: approximately 8 million, annually 1100–1200 new cases of paediatric cancer:

- 13 regional centres of paediatric oncology which consist of 18 wards, all units collaborate within Polish Paediatric Leukemia/Lymphoma Study Group and Polish Paediatric Solid Tumors Study Group
- a total of 688 beds
- 130 specialists in paediatric oncology/hematology, 50 additional on training
- five paediatric BMT Units, approximately 130 transplants/year

Financial resources for paediatric oncology in Poland:

- National Health Fund (health insurance)—diagnostics, therapy, hospitalization, medicines
- National Program Against Cancer (granted by the parliament)—education, standardization of diagnostic procedures, nationwide prophylaxis programs, programs for modernization of medical equipment
- Parents organizations and charity foundations (mainly social costs, and individual centre’s support to buy equipment, devices, renovations
- Ministry of Health for some highly specialized procedures
- Ministry of Science for research in medicine

### 2. What are the key issues for paediatric oncology in delivering care and research in Poland?

The main issue is to provide resources for complete diagnosis and therapy of children with cancer. Generally, all paediatric procedures are priced by the National Health Fund below the real costs. Thus, some diagnostic cost of care related for example to molecular or immunological examinations we try to cover in part using funds received for research.

### 3. What are the sources of funding for your paediatric oncology research. What are your views on the balance between public and commercial sources of income?

Research is basically founded by Universities (internal grants)—very limited subsidy to each research unit (the amount of money depends on the research activity of the unit measured as a publications score in last year). More ambitious research project can enter a competition organized by the Ministry of Science twice a year. Occasionally, some possibilities to get a financial support come from non-governmental foundations for specific projects. The commercial sources of income for research in Poland almost do not exist.

### 4. The state of patient information in Poland for childhood cancers

Patient information for childhood cancer in Poland is provided on the local level (staff of a paediatric oncology ward is mainly responsible and local parent’s organizations are also involved in it). At the central level there several web pages and booklets provided by non-governmental organization or societies (ex. Polish Union of Oncology—http://www.puo.pl).

### 5. What effect has European level funding (Framework Programme etc.) had on European paediatric oncology?

Not specified.

### 6. Key areas to be addressed by the Commission in the next 5 years

In the case of the multicentre network it can give the opportunity for harmonization and standardization especially for new EU Member States. The key issue for the next 5 years in my opinion is to facilitate the implementation of academic clinical trials in each country. In the case of academic trials based on international, multicentres cooperation, the main coordinator should have an opportunity to apply for funding from European Commission (or specific Agency) to cover all expenses related to implementation and to conduct the trial in each participating country.

## Portugal

**Table:** 

Name	Maria João Gil-da-Costa
Country	Portugal
Position	Paediatric Oncologist Consultant/Responsible for Brain Tumour Patients
Institution	University Hospital S. João—Porto

### 1. How is paediatric care and research delivered in Portugal?

We have four Paediatric Oncology Units (POU) in Portugal, one in Lisbon—Paediatric Department of the Portuguese Cancer Institute (South), two in Oporto (North) and one in Coimbra—Paediatric Hospital (Centre). Since January 2008, the Health Authorities decided to do some differential referentiation in the North of the country to the two POUs, because of the human resources and facilities of the two hospitals. As so, the POU located at the Portuguese Cancer Institute Paediatric Department receives all leukaemia/lymphoma patients and the POU located at the University Hospital S. João Paediatric Department receives all Brain Tumour patients. Both units are able to treat all the other types of solid tumours.

We have about 300–350 new cancer patients per year (0–15 years), but in some POU, namely in Oporto, we are now treating adolescent patients until 18 years old, as a consequence we expect more patients in our units to be treated by paediatric oncologists.

We have a Paediatric Haematology and Oncology Society integrated in the Portuguese Paediatric Society (Chairwoman—Dr Filomena Pereira—POU Lisbon) and a College of Paediatric Oncology, which is now responsible for the definition of the standards of training for new paediatric oncologists (Chairwoman—Dr Lucília Norton—POU from Cancer Institute of Oporto). We do not have any Portuguese Treatment Protocol and we do not follow exactly the same treatments in all POUs. We use to follow SIOP’s protocols, EORTC leukaemia protocols (namely in Oporto) and some COG, Dana-Faber Cancer Institute and HIT protocols. At the moment we only register patients in EORTC leukaemia protocols (Oporto), SIOPEN protocols (Lisbon) and some more rare isolated patients from the various POUs. We are trying to take part in the new trials, namely for brain tumours and renal tumours, but we have many difficulties, specially related with insurances, funding and logistic facilities.

We are going to try to find support from some Foundations, like Champalimaud, Calouste Glubenkian and Inês Botelho. In Portugal we have a Cancer Parents and Friends Association ‘Acreditar’, witch is specially oriented for families and children supports and not for clinical or basic research. The Hospitals themselves do not have enough funding for research.

### 2. What are the key issues for paediatric oncology in delivering care and research in Portugal?

The Portuguese National Oncology Plan is very poor in what concerns paediatric oncology. Portugal is now developing a document that defines the minimal standards of care for Oncologic Patients, but it still not oriented for Paediatric Patients. I sent the new document of SIOPE about the Standards of Care to the members of the Portuguese Paediatric Haematology and Oncology Society, and to the Portuguese Health Authorities, so that we are able to follow, at least in part, the SIOPE recommendations in our POUs. I think we can only become formal partners of the clinical trials, if we can access to some common platform, namely with the umbrella of SIOPE, for funding and logistic facilities.

### 3. What are the sources of funding for your paediatric oncology research. What are your views on the balance between public and commercial sources of income?

As I said before the funding sources are very scarce, particularly at the public level; now we have more economic difficulties and our institutional management teams have difficulty in understanding the importance of including patients in international trials. Finding funding is also a problem when considering participations in international congresses and meetings, since the pharmaceutical laboratories, which use to be ours funders, are now cutting on their expenses.

### 4. The state of patient information in Portugal for childhood cancers

All the POUs have some ‘booklets’ with information for parents about general care during the treatments and ‘Acreditar’ developed many books, some of them by translation of international ones with permission in the platform of ICCCPO. Some years ago a site named ‘http://www.oncologiapediatrica.org’ started to be developed, in collaboration with ‘Acreditar’ and the Institute of Cancer, were some general and specific tumours information has been included.

### 5. What effect has European level funding (Framework Programme etc.) had on European paediatric oncology?

Not specified.

### 6. Key areas to be addressed by the Commission in the next 5 years

If SIOPE effectively has the objective to extend the ‘state of the art’ of care for all European paediatric patients, and since small countries have many difficulties in creating the necessary logistic facilities and funding to do so by themselves, it is necessary that SIOPE itself creates a common platform, preferably built with European funds, that answers this urgent need.

## Portugal

**Table:** 

Name	Lucília Norton
Country	Portugal
Position	Head of Paediatric Department
Institution	Instituto Português de Oncologia do Porto (IPO Porto)

### 1. How is paediatric care and research delivered in Portugal?

In Portugal there are four units: Two units in Oporto, one in Coimbra and one in Lisbon. There isn’t yet a National Pediatric Oncology Network. In the Department of Pediatric Oncology of the Institute of Cancer in Oporto we have 23 rooms, 11 are individual, 8 of which have HEPA filters and positive pressure. We have access to all Paediatric Oncology Subspecialties, most of them in another Institution (Hospital S. João) on the other side of the street. We also have all the labs we need. Every Centre develops its own research. In the ‘Instituto Português de Oncologia’ in Oporto there is a research centre (Basic sciences) which works closely with the clinicians. We are integrated in the CLG–EORTC (acute leukaemias). We are trying to participate in the SIOPEN (Neuroblastoma), RTSG (Wilms) and in other Cooperative Groups. We have in our Institution a Data Management Centre. So, I think we have here what is needed to provide appropriate care and develop research.

### 2. What are the key issues for paediatric oncology in delivering care and research in Portugal?

- To develop a better network with our Primary Care System.
- To get all the Centres participating in cooperative trials.

### 3. What are the sources of funding for your paediatric oncology research. What are your views on the balance between public and commercial sources of income?

The funding of our Hospitals—clinic and/or research—is mostly public. Recently there are some private foundations who provide funding for specific projects. I think, we should try to get public funding for the most important projects but it is important to get some additional help from the private Institutions.

### 4. The state of patient information in Portugal for childhood cancers

All the parents, and the children when appropriated, are informed of the disease and treatment. An Institutional Informed Consent is obtained before chemotherapy and before every diagnostic and therapeutic procedure not included in the initial Informed Consent. From research there are specific informed consents approved by the National Research Ethics Committee (CEIC). I don’t think there are gaps in this Institution in the information.

### 5. What effect has European level funding (Framework Programme etc.) had on European paediatric oncology?

Not specified.

### 6. Key areas to be addressed by the Commission in the next 5 years

The Commission should endeavour to include all the paediatric patients in cooperative trials.

## Portugal

**Table:** 

Name	Filomena Pereira
Country	Portugal
Position	Chairwoman of Paediatric Haematology and Oncology Society/Portuguese Paediatric Society
Institution	LISBON POU, Paediatric Department of the Portuguese Institute of Oncology in Lisbon

### 1. How is paediatric care and research delivered in Portugal?

In Portugal there are four paediatric oncology units. There is no research at a national level.

- Portuguese Institute of Oncology—Lisbon, receives 160 new patients/year, half of national patients; we receive patients from the south of the country, Azores, Madeira and PALOP. It has 22 beds; full time staff—seven paediatric oncologists, one paediatric neuro-oncologist; part time staff—one neuro-oncologist, three paediatric surgeons; participates in SIOPEN; research has been funded mainly by the Institute itself.
- Portuguese Institute of Oncology—Oporto.
- Pediatric Hospital—Coimbra.
- Hospital S. João (Oporto).

### 2. What are the key issues for paediatric oncology in delivering care and research in Portugal?

The key issues for developing Pediatric Oncology in Portugal currently are:

- To have four units that does’t follow exactly the same protocols, namely of leukaemia treatment (in the other pathologies, the SIOP protocols are used).
- Improve cooperation with the local health-care centres (hospitals and community centres, family doctors), necessary due to the wide geographic reach of each of the units; The Lisbon POU has a direct relationship with peripheral paediatric centres for sharing palliative and terminal ill patients care.
- Register and randomize patients in European multicentre clinical studies.

### 3. What are the sources of funding for your paediatric oncology research. What are your views on the balance between public and commercial sources of income?

In Portugal the funding for research comes from:

- One on one private initiative (we ask for help and money from Foundations and Companies, some of them pharmaceutical);
- Internal resources from the Institutes/Hospitals.

We think that the Ministry of Health should finance these activities, at least assuring the minimum amounts for insurance, in order to allow us to have no basic problems in entering our patients in the European ongoing state-of-the-art clinical studies. It is crucial that we gather efforts to collect experience in such rare diseases.

### 4. The state of patient information in Portugal for childhood cancers

Information is given to patients in the following form:

- Portuguese Institute of Oncology—Lisbon;
- Personal interviews with the multidisciplinary staff;
- Several leaflets on admission, discharge, etc. (currently preparing a caregiver ‘welcome and how-to’ handbook);
- A care giver handbook for children in palliation;

Informed consent is requested from parents; we think that adolescents (>14 years old) should be addressed differently and they should be the ones to sign their consent form.

### 5. What effect has European level funding (Framework Programme etc.) had on European paediatric oncology?

Portugal has been participating in the SIOPEN studies, one of which (HRNBL-1) was in part funded by a grant from the E.C. Otherwise, the study would have been unable to be launched!

### 6. Key areas to be addressed by the Commission in the next 5 years

For small countries like Portugal, that don’t have a national organization for fund raising and bureaucratic issues, the latest European directive has made things very very hard to get started. Also, we don’t understand why a protocol needs to be approved at each and every country by both Ethical Commissions and Drug Regulatory Agencies, when it could all be done once under a ‘European umbrella’. It would save so much time and money….

## Serbia

**Table:** 

Name	Dragana Janic
Country	Serbia
Position	Head of Department of Hematology and Oncology, Professor of Paediatrics
Institution	University Children’s Hospital School of Medicine, University of Belgrade

### 1. How is paediatric care and research delivered in Serbia?

Paediatric care in our country is publicly available to all children aged <18 years. All paediatric care facilities are a part of government funded, primary to tertiary level centres and hospitals. Also, all existing centres for paediatric haematology and oncology are united through a subsection of Serbian Medical Society (a.k.a. Intersection Board of Paediatric Haematology and Oncology of Serbian Medical Society). Individual centres and hospitals do not have dedicated funds for research.

### 2. What are the key issues for paediatric oncology in delivering care and research in Serbia?

Key issue is lack of staff. Standards and normatives for facilities and staff are not specifically defined and are not different compared to general paediatric wards. There is a need for education and employing psychologists and medical secretaries who are usually not provided. Data management is not recognized. Participation in clinical trials is not supported. Research in the field of paediatric oncology is not supported at all by the funds.

### 3. What are the sources of funding for your paediatric oncology research. What are your views on the balance between public and commercial sources of income?

The only sources of public funding for research are grants of the Ministry of Science. Paediatric oncology is applying with all other medical disciplines for the grants. Commercial sources are not often used in our country. To my opinion we should use more commercial sources of funding. The research should be planned according to GCP and ethical standards which should be appropriately stated in all medical publications.

### 4. The state of patient information in Serbia for childhood cancers

Information is given by paediatric oncologists themselves. There are only few published patient information booklets and our national parents’ society is currently working to produce more.

### 5. What effect has European level funding (Framework Programme etc.) had on European paediatric oncology?

Not specified.

### 6. Key areas to be addressed by the Commission in the next 5 years

The key issue is giving more support to academic trials in paediatric oncology.

## Slovakia

**Table:** 

Name	Judita Puskacova
Country	Slovakia
Position	Chair
Institution	Division of Pediatric Hemathology and Oncology in Slovakia

### 1. How is paediatric care and research delivered in Slovakia?

There are about 5,000,000 inhabitants in Slovakia, with about 160–180 new paediatric patients with malignancies yearly. Since 1992 the paediatric cancer care for children from birth to 18 has been centralized in the three oncology centres: Bratislava with 50% of patients (for West part of Slovakia), Banská Bystrica with 25% (Middle part) and Kosice with 25% of patients (East part of Slovakia). The biggest centre in Bratislava has two departments with 34 beds together and three outpatients clinics (one clinic for patients on treatment and two clinics for patients after treatment, they are followed up until reaching 19 years of age). This clinic takes part in the pre-graduate paediatric oncology education for medical students, students of nursing and public health service students. The sub-department of paediatric oncology at the Slovak Health University is a part of this clinic. It covers postgraduate education for physicians and middle medical staff of whole Slovakia.

All tree Slovak centres are incorporated in the Division of Pediatric Hemathology and Oncology of the Slovak Medical Society, cooperating well and coordinating the medical care, using common standards. The treatment is according to protocols of SIOP, I-BFM and some other European and American oncology groups and is the same in all the three paediatric oncology centres. Centres integrate to international studies gradually. The number of paediatricians with specialization in paediatric oncology has increased.

Hematopoietic stem cells transplantations are performed for whole Slovakia at the Transplantation unit of Pediatric clinic of the University Children Hospital in Bratislava. Establishment of new examinational methods and improvement of the present ones (18 FDG-PET, flow cytometry, methods of molecular genetics and cytogenetics) enabled significant improvement of diagnostic procedures. Assessment of minimal residual disease in leukaemia is developed with cooperation of the Department of Genetics at National Cancer Institute at present. The anticancer therapy late effects study is solved in multilateral cooperation among the Medical Faculty and several specialized paediatric departments.

### 2. What are the key issues for paediatric oncology in delivering care and research in Slovakia?

As for research it is only at developmental stage. It is funded mostly from public sources, partly with Slovakian Ministry of Health and partly from grants.

### 3. What are the sources of funding for your paediatric oncology research. What are your views on the balance between public and commercial sources of income?

Slovakia as one of the new members of the EU has been implementing European law and regulations, which, of course, include health care. The principles of GCP management are embraced in the EU directive 2001/20, which has been valid in Slovakia since 2006. In the paediatric oncology it is essential that such attempts and/or happening are entailed. Key issues of paediatric oncology are to provide as good care as it is in another countries of West Europe, to become an active members of European most modern studies for paediatric oncology. As for research so far we can provide our samples to most developed European project and to become cooperators.

### 4. The state of patient information in Slovakia for childhood cancers

As for patient information about cancer: this is provided mostly by skilled paediatric oncologists and it is part of whole process of informed consent. As for gaps: we do not have standards and we do not have any training for physicians about informing patients and also psychologists could be more involved in this process.

### 5. What effect has European level funding (Framework Programme etc.) had on European paediatric oncology?

The EUROCARE-3 study from the years 1990–1994 showed improvement of survival in all countries (European average was 71, 8%), but Slovakia with 5-years OS 63, 1% was still on the last but one position. Results from the period 2000–2003 from all oncology centres in Slovakia are better in all parameters than those in east European countries in the EUROCARE-3 study. 10-years survival 65% in 1992–2001 and 5-years survival 77% in 2000–2005 as well as results in particular diagnoses were at the Clinic of Pediatric Hematology and Oncology in Bratislava better than results for whole Slovakia published in the EUROCARE study-3 too. Superior health-care organization, new and more effective examinational techniques and therapeutic methods, better skills of paediatric oncologists and surgeons as well, all that plays the important role in this improvement.

### 6. Key areas to be addressed by the Commission in the next 5 years

Not specified.

## Slovenia

**Table:** 

Name	Janez Jazbec
Country	Slovenia
Position	Head of the department
Institution	University Medical Centre Ljubljana, Children’s Hospital, Department of Haematology and Oncology

### 1. How is paediatric care and research delivered in Slovenia?

In Slovenia (approximately 2 million inhabitants) there is one paediatric oncology centre. No national network. Research is mainly funded through the Ministry of higher Education, Science and Technology on the basis of applied projects. Relatively minor source of income for research comes from other sources (foundation for children with cancer). Practically no income from commercial sponsors.

### 2. What are the key issues for paediatric oncology in delivering care and research in Slovenia?

The key issue is to keep the high level of expertise in the field of paediatric oncology, despite relatively low number of patients. Main solution is to be involved in international collaborative studies and projects and to be active member of international learned societies (SIOP, BFM…)

### 3. What are the sources of funding for your paediatric oncology research. What are your views on the balance between public and commercial sources of income?

See answer no. 1.

### 4. The state of patient information in Slovenia for childhood cancers

Due to language barrier and small population our patients may be in deprivileged position to other population where huge amount of information can be found in native tongue at various sources (www) Our patients must rely on information gathered from treating physicians. There are a couple of booklets written on the topic of childhood cancer, treatment and long-term sequelae in Slovenian. Booklets were written by domestic experts and published by Slovenian Foundation for Children with cancer. Another valuable source of information is National organization of parents of children with cancer, through parents who have already gone through, interact with the families with newly diagnosed child.

### 5. What effect has European level funding (Framework Programme etc.) had on European paediatric oncology?

There has bee no effect observed on the level of routine diagnostics and treatment of children with cancer in Slovenia so far.

### 6. Key areas to be addressed by the Commission in the next 5 years

The major problem, that emerged is involvement in multi-central collaborative studies (which is considered as the best level of treatment of children with cancer as you may agree), where several administrative obstacles can severely delay involvement in such projects.

## Spain

**Table:** 

Name	Adela Cañete
Country	Spain
Position	Attending Physician
Institution	H. U. La Fe Pediatric Oncology Unit

### 1. How is paediatric care and research delivered in Spain?

Clinical care is giving mostly through the National Health System in all the regions (Comunidades Autonomas: CA) in Spain in Pediatric Oncology units in main hospitals. There are some few private hospitals with Pediatric Oncology units too. Care is multidisciplinary in most of the places. There are a total of 20–25 ‘so called’ ‘Pediatric Oncology units’ because for political reasons every CA has to have at least one Pediatric Oncology unit in order to close care to patient’s home. Out of those 20–25 Units, half of them at most will see more than 50 patients per year. Bigger units (>100 patients per year) are in Valencia, Barcelona, Madrid, Sevilla. Research is not so well organized, only big Pediatric Oncology unit have programs in research (Valencia, Bilbao, Madrid, Barcelona). It is funded through public and private competitive grants, not specifically designed for paediatrics. There is a new network created to develop phase I and II trials, with the participation of big Units in the country. Psycooncological and social care is also provided, generally through parent association. Education needs are also provided with the help of the Education Ministry.

### 2. What are the key issues for paediatric oncology in delivering care and research in Spain?

In delivering care, the key issue would be to classify the Unit in different levels of care according to complexity. In research it would be to potentiate clinical research and develop more translational and basic research. For that, we need a recognition of the need from the political level.

### 3. What are the sources of funding for your paediatric oncology research. What are your views on the balance between public and commercial sources of income?

Most of the funding in public, through competitive grants (usually against adult oncology). Private is scarce. Charities (including parent associations) are more directed to provide social and fun care than to help research. Clinical research is not one of their fields of interest.

### 4. The state of patient information in Spain for childhood cancers

Parents are very keen on internet and nowadays they surf the net without any advice of help, making some communication and information troubles, misunderstandings. Parents and children are informed by the doctors (usually the responsible doctor) but also parent association have their own uncontrolled and unsupervised medical information, which may create problems.

### 5. What effect has European level funding (Framework Programme etc.) had on European paediatric oncology?

I think not so big impact till now.

### 6. Key areas to be addressed by the Commission in the next 5 years

The efforts should be made in several directions:

1. to improve care: funding clinical and translational research, access to new drugs and more knowledge about old drugs in kids,
2. to balance care all over Europe, helping to improve nationally. The EU can force the member states to improve the weak areas in care and research.

## Sweden

**Table:** 

Name	Lars Hjorth
Country	Sweden
Position	Consultant, M.D., Ph.D.
Institution	Paediatrics, Clinical Sciences Lund
Name	Gustaf Ljungman
Country	Sweden
Position	Consultant, M.D., Ph.D. Associate Professor
Institution	Paediatrics, Clinical Sciences Uppsala

### 1. How is paediatric care and research delivered in Sweden?

In Sweden, with a population of about 9,000,000 people, we get approximately 250 new childhood cancer cases below 15 years of age per year. If we count up to 18 years it is slightly more than 300/year. For this we have six paediatric oncology units; Stockholm, Gothenburg, Lund, Uppsala, Linköping and Umeå. Within the Swedish Paediatric Society we have a Section for Paediatric Haematology and Oncology that is the decision making body for the country. The board is made up of representatives for the working groups, the registry and the local hospitals outside of the centres. The working groups are: the Swedish Childhood Leukaemia Group, the Swedish Childhood Solid Tumour Group, the Swedish Childhood Brain Tumour Group, the Swedish Working Group for Long-Term Follow-Up after Childhood Cancer and the Working Group for Paediatric Haematology. More than 90% of the research is funded outside of the government, through donations etc., with the Swedish Childhood Cancer Foundation being one of the major contributors.

### 2. What are the key issues for paediatric oncology in delivering care and research in Sweden?

Key issues in Sweden are the same as in several other countries, I would believe. Lack of funds, lack of experienced staff including doctors due to not educating enough people. We have hired doctors from other non-Scandinavian countries which is good for us but bad for the countries that lose their staff. On the whole, however, we still feel that we can deliver the care that we want to almost all our children and adolescents. It is some of the resources around the care, e.g. reporting information to study centres, ethical applications, costs associated with following GCP, follow-up facilities/programmes etc. that are not funded today. For research purposes we would like to see more money being allocated by the government. The lack of experienced clinical staff sometimes has the ironic effect that research money already received cannot be used since the staff is ‘forced’ to work clinically instead of with research.

### 3. What are the sources of funding for your paediatric oncology research. What are your views on the balance between public and commercial sources of income?

Today we get very little commercial support for research. We have previously been involved in very few industry sponsored studies and therefore have received very little money that way. For example, in Lund we have joined only one study (IGF1-R blocker) in all the years I have been active. Most other studies are non-industry sponsored and provide us with only costs and no income. We would like to see the government take a larger responsibility for supporting us with money for all the necessary work involved in all the studies we take part in but at present do not get paid for. It is not OK that donated money goes to pay for things that are really part of providing the children with the necessary and appropriate care involved when treating children according to existing protocols. If this is not corrected for, we may have to decline participation in future studies due to a lack of resources and treat the children outside of trials, which is known to produce inferior results.

### 4. The state of patient information in Sweden for childhood cancers

Most information concerning childhood cancer in Sweden is provided thanks to the Swedish Childhood Cancer Foundation. Doctors and nurses, sometimes within the scope of one of the existing Working Group, have put together the material and the Swedish Childhood Cancer Foundation has the paid for printing and seen to the distribution. A lot of this information is also available on their web page. I feel that we are not in total control over how recent and updated the information is, simply because we do not have a system for this and no one designated to do it. Much of this work is done outside of office hours without correct reimbursement.

### 5. What effect has European level funding (Framework Programme etc.) had on European paediatric oncology?

Previous European funding for paediatric oncology is not well known by me.

### 6. Key areas to be addressed by the Commission in the next 5 years

The recently approved ENCCA and PanCareSurFup projects will together have close to 18,000,000 Euro over the next 4–5 years and spin-offs from this could very well be funded at European level, provided we can give the right input at the right level within the EU. If a solid basis and structure for paediatric oncology could be set up within Europe, this would lead to better use of money allocated and a quicker way forward thanks to synergies in collaboration and complementary work being done. The ENCCA is a start for generic paediatric oncology purposes and should be used as a stepping stone forward. If much of the paediatric oncology research and care for patients work is ‘channelled’ through one instance, it is reasonable to believe we would reach success in a quicker and more complementary way. Hopefully PanCareSurFup can lead to future studies and projects for all the different survivorship issues.

## Turkey

**Table:** 

Name	Tezer Kutluk
Country	Turkey
Position	MD PhD; Professor of Pediatrics
Institution	Hacettepe University Faculty of Medicine

### 1. How is paediatric care and research delivered in Turkey?

Modern Pediatric Oncology started in late sixties in Turkey. Now there are 31 paediatric oncology unit. Most of them are comprehensive centres which has paediatric oncology, paediatric surgery, pathology, diagnostic and other departments. There are 91 paediatric oncologist. Pediatric Oncology is a subspecialty which needs a 3-year training period. 68 centres are entering data to national paediatric cancer registry and 44 of them are paediatric oncologic centres or units at different size. Turkish Pediatric Oncology Group (TPOG) was established in 1997. This a platform for sharing the knowledge and expertise. The organization has bi-annual meeting with a large participation from its members. Each University Hospital has a research fund and members of that university has right to apply for a grant. The other option is The Scientific and Technical Research Council of Turkey (TUBÝTAK). However it is also open to other disciplines in addition to Medical Sciences. Industry sponsored research is limited in paediatric oncology population. Although there is a possibility of funding the research by other NGO’s and sources, this is a limited source.

### 2. What are the key issues for paediatric oncology in delivering care and research in Turkey?

The level of clinical care is quite good. The quality of the medical training at undergraduate and postgraduate level is also very well. Each year 2500–3000 new cancer cases are expected in Turkey. Access to care is at good level. On the other hand basic and translational research is limited because of the limited funding.

### 3. What are the sources of funding for your paediatric oncology research. What are your views on the balance between public and commercial sources of income?

The source are identified in question 1. Both University funds and TUBÝTAK are limited and because of that there is less interest in lab research. Commercial sources are also limited. Recently, 2 years ago, Turkish Pediatric Oncology Group started a ‘research grant program’ with its limited financial sources. The grant is allocated for three research project with 10 000 USD to each.

### 4. The state of patient information in Turkey for childhood cancers

Patients are informed by the treatment centres. Since most of the knowledge on the internet is in English, there is need for patient information. Turkish Association for Cancer Research and Control is organizing Cancer Patient forums annually for adult and paediatric cancer patients and relatives since 2006. Some local NGO’s are also providing patient information.

### 5. What effect has European level funding (Framework Programme etc.) had on European paediatric oncology?

Not specified.

### 6. Key areas to be addressed by the Commission in the next 5 years

Paediatric cancers are rare diseases compared with adult cancers and this is a limiting factor for clinical trials. The support for rare disease and orphan drugs by Commission will be helpful.

## United Kingdom

**Table:** 

Name	Kathy Pritchard-Jones
Country	UK
Position	Consultant Paediatric Oncologist (Gt Ormond St hospital), Professor of Paediatric Oncology (University College Hospital) & Programme Director for Cancer, University College London Partners
Institution	As above

### 1. How is paediatric care and research delivered in the UK?

Twenty-one principal treatment centres (PTCs) organized into a national network to deliver standardized treatment guidelines and clinical trials to a uniform standard across the country (includes UK and Republic of Ireland where there is one PTC in Dublin). The PTCs have been in existence since 1977, when the UK Children’s Cancer Study Group (UKCCSG) formed. There has been some reduction in the number of centres since that time (from 22 to 21, with the merger of Leicester into Nottingham) and there is likely to be further rationalization through partnership of smaller centres into one principal centre and a satellite paediatric oncology ‘shared care’ unit (POSCU) in some regions where two small PTCs are close to each other.

Each PTC works to a greater or lesser extent with children’s local hospitals (POSCUs) for more local provision of mainly supportive care and outpatient chemotherapy elements of treatment—the extent of devolution of care varies enormously around the country, partially but not wholly dependent on geography (e.g. the South West, where travelling times to the nearest PTC at Bristol are >2 hours, have devolved most inpatient chemotherapy back to a small number of POSCUs, others, such as Newcastle, bring all patients back to the centre for supportive care). The PTC-POSCU relationship is a dynamic one and is influenced by ongoing implementation of the national cancer ‘Improving Outcomes Guidance’ (IOG) for children and young person’s cancers issued by the NICE. The guidance was published in 2005, the ‘measures’ against which compliance of PTCs and POSCUs will be assessed, was published in late 2009 and the peer review of compliance will take place during late 2010/early 2011 by ‘self assessment’ of each unit against the written standards. This will be followed up by external peer review in 2012 (most likely, though the new government could change the timescales). The NICE-IOGs are a way to set standards for deliver of cancer care in all cancers. However, they only apply to England–Wales, Scotland and Northern Ireland have their own equivalents, The Republic of Ireland is of course a separate country that has to be bound by Irish Dept of Health laws and initiatives.

The above describes the ‘cancer networks’ that are all about setting and implementing standards of care to improve outcomes. There is a parallel cancer research network organization that facilitates and monitors entry into clinical trials. On top of this, there is the ability for centres to bid to become an Experimental Cancer Medicine Centre, where there are additional resources provided for running early phase trials. Currently, only four CCLG PTCs are designated paediatric ECMC centres.

Cancer has been in the lead for organizing networks with agreed standards of care and parallel clinical networks in the UK, since the launch of the National Cancer Plan in 2000. However, other areas of medicine are now being organized with a similar approach. Hence, the resources to support clinical trials at a local level (i.e. towards data management, research nurse and trial coordination costs) are now increasingly meant to be sought from the ‘Comprehensive Clinical Research Networks’ that are resourced to support all types of clinical research within the NHS by region, rather than specific cancer research networks (although the latter still exist for the moment).

In parallel with these changes, the national childhood cancer group (Children’s Cancer and Leukaemia Group, CCLG, formerly known as UKCCSG) has undergone a major change during 2009/10. It has now been split into four domains of activity which were all previously under the CCLG:

Clinical trials are now overseen by a National Cancer Research Network (NCRN) Paediatric Clinical Studies Group—formed in Dec 2009. This covers most paediatric cancers, with eight specific subgroups, but some cancers.

### 2. What are the key issues for paediatric oncology in delivering care and research in the UK?

Not specified.

### 3. What are the sources of funding for your paediatric oncology research. What are your views on the balance between public and commercial sources of income?

Not specified.

### 4. The state of patient information in the UK for childhood cancers

Not specified.

### 5. What effect has European level funding (Framework Programme etc.) had on European paediatric oncology?

Not specified.

### Key areas to be addressed by the Commission in the next 5 years

Not specified.

## United Kingdom

**Table:** 

Name	Bruce Morland
Country	UK
Position	Paediatric Oncologist
Institution	Birmingham Children’s Hospital

### 1. How is paediatric care and research delivered in the UK?

There is a network of 21 Principal Treatment Centres (PTC) throughout the UK and Ireland. All Centres are linked professionally through the Children’s Cancer and Leukaemia Group (CCLG). Funding for clinical care is provided for by the NHS in England, Scotland and Wales and by a separate Health Board in Ireland. Research within the network is funded through the National Institute for Health Research (NIHR) which provides infrastructure support (research nurse, data management etc) through the auspices of the National Cancer Research Institute and through local Comprehensive Research Networks (CLRN). Clinical research funding is heavily weighted to supporting trials registered on the NIHR portfolio. Some children will receive part or all of their treatment in shared care units (POSCU), linked to one or more PTCs. The quality of care provided by networks of PTCs and POSCUs is undergoing a process of external peer review during 2010/11 underpinned by published national guidelines.

### 2. What are the key issues for paediatric oncology in delivering care and research in the UK?

Uncertainty about the future implication of public spending cuts on NHS funding. There is no reason to believe that children’s cancer care will be protected from funding pressures. The national Peer Review will flag up gaps in service but there is no additional commitment to funding these gaps. Historical reliance on charitable funding of core service support staff (CLIC Sargent social work, MacMillan nurses etc) is also subject to financial pressure due to reduced charitable giving in the current climate. Research has been coordinated and centralized in the UK for many years. Infrastructure changes have moved paediatrics alongside the adult cancer model. Whilst this is entirely appropriate it does mean that research for children now ‘competes’ at a much more visible level with adult cancer and we need to be sure that children still receive a fair slice of the funding cake.

### 3. What are the sources of funding for your paediatric oncology research. What are your views on the balance between public and commercial sources of income?

The vast majority of research funding comes from Government and research charities (Cancer Research UK, Medical Research Council, Leukaemia Research Fund in the main). Very little funding comes from commercial sources, mainly some early phase drug development work. This balance seems to work well especially as there is a strong history of very good funding support to children from the medical charities. Commercial opportunities will always be limited but could probably be enhance further.

### 4. The state of patient information in the UK for childhood cancers

Generally very good and of a very high standard. The majority of work has been led by CCLG who have a publications committee and a very strong association with patients and parents. They are a shining example of what can be achieved!

### 5. What effect has European level funding (Framework Programme etc.) had on European paediatric oncology?

There have been few projects. Some isolated areas of funding mainly in the area of translational/pharmacology/drug development. Recent funding of the ENCCA NoE is welcome.

### 6. Key areas to be addressed by the Commission in the next 5 years

Targeting funding to provide infrastructure support in the running of pan-European trials is essential and to help with the bureaucracy-busting hampering so much good will to collaborate within Europe. There is a real and urgent need to develop practical method for undertaking pan-European clinical trials in rare diseases. Many childhood cancers fall into this area. The ability to investigate methods of centralized trial approvals across EU national boundaries for rare diseases is essential.

## United Kingdom

**Table:** 

Name	Michael Stevens
Country	UK
Position	Professor of Paediatric Oncology
Institution	University of Bristol

### 1. How is paediatric care and research delivered in the UK?

More than 90% of children with cancer and leukaemia (CCL) in the UK are referred to one of 21 specialist centres (Principal Treatment Centres—PTCs) linked to the UK Children’s Cancer and Leukaemia Group (CCLG). In addition, there are an increasing number of designated centres (PTCs) for the treatment of Teenagers and Young Adults with cancer that are co located with the paediatric centres. In this way most parts of the country now have designated specialist units that provide care across the entire Children’s—Teenage—Young Adult (CTYA) age range (now defined by government policy as being from 0–24 years). In addition to the CCLG (http://www.cclg.org.uk), there is another organization which provides a focus for professionals involved in the care of TYA patients with cancer (TYAC—http://www.tyac.org.uk). Neither of these organizations now develop or run clinical trials but they represent the basic coordinating and professional structure for the paediatric and TYA cancer community.

Until March 2010, CCLG was the largest national organization responsible for the development and management of clinical trials, particularly in solid and CNS tumours, for children and TYA patients—although other organizations were responsible for the management of, for example, some leukaemia and bone tumour studies. Since then, the Cancer Research Clinical Trials Unit (CRCTU) at the University of Birmingham has become responsible for the majority of CCL trials in the UK. CRCTU will also assume responsibility for the management of the next generation of national leukaemia studies.

**Figure 14:**
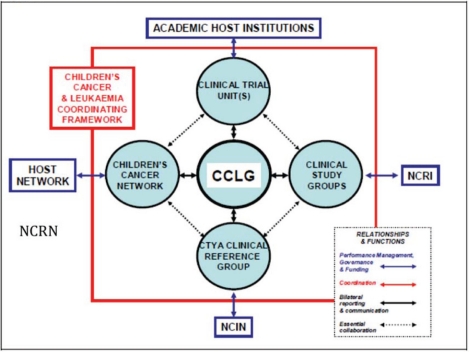
Organisation of childhood cancer research in UK.

The majority of funding for solid tumour and brain tumour trials is provided by grants from Cancer Research UK (the largest cancer research charity in the country) but resources for leukaemia studies are provided by another national research funder (Leukaemia and Lymphoma Research) and various other funders contribute to smaller studies. There is a limited amount of pharma support for early phase studies most of which are run in conjunction with the ITCC (http://www.itcc-consortium.org). Resources for the 21 treatment centres which support CCL studies are currently under review. A recent reconfiguration of funding support is being implemented and oversight of the CCL network (funding, governance and performance management) will now be undertaken by the National Cancer Research Network (http://www.ncrndev.org.uk). A separate structure has been created to support the development and implementation of the CCL portfolio. This aligns with arrangements for adult cancer in the UK and there is now a Clinical Studies Group for CCL trials within the NCRN. Finally, resources for CTYA cancer registration and for observational and epidemiological studies are now overseen by a CTYA clinical reference group within another new structure, the National Cancer Information Network (NCIN—http://www.ncin.org.uk). The overall complexity of structures involved with the support and delivery of care to children and young people with cancer in the UK is shown in Figure 14:

The leaders of each of the different ‘domains’ shown in the diagram meet regularly as the CCL Chairs Forum to coordinate their activities.

### 2. What are the key issues for paediatric oncology in delivering care and research in the UK?

The key issue for the paediatric oncology has been the significant reconfiguration of operating structures described above. There are also issues about different arrangements for funding support within different areas of the United Kingdom (rules which apply in England do not necessarily apply in Scotland, Wales and Northern Ireland). It is too early to know how much these changes will facilitate or restrict activity but it is believed that clinical trial support and governance should be improved and that funding for the network of treatment centres will become more secure. These changes affect research structures and research resources but the funding of care is unaffected—the challenge to this will come from the ability of the national economy to continue to support the level of care which has been defined in a set of nationally agreed standards for the delivery of care to CTYA with cancer. These standards define the resources necessary to deliver care both in the Principal Treatment Centres and the shared care units with which they work.

### 3. What are the sources of funding for your paediatric oncology research. What are your views on the balance between public and commercial sources of income?

There is a well established source of research support funding available through the National Health Service but this has previously not been made available to CCL centres in a consistent way. This is likely to change and should improve the stability of resources to support participation in clinical trials. The support of the trials themselves will need to be funded by competitive grant applications. Commercial income is likely to remain a small component of funding for paediatric oncology research but will be expected for some early phase trials and may increase with the development of paediatric investigation plans for licensing requirements of new drugs in Europe.

### 4. The state of patient information in the UK for childhood cancers

CCLG provides an excellent range of publications for children, young people and their families. These publications are made available (currently free of charge) to all CCL treatment centres and are available to the general public on its website (http://www.cclg.org.uk/families/). CCLG also publishes a national magazine for families (http://www.cclg.org.uk/contact/index.php?2id=10) which includes regular items about cancer treatment and support. Other cancer charities provide additional information and many CCL units supplement this with their own local information for patients. Resources specifically for TYA are also provided by a number of organizations, most notably by the Teenage Cancer Trust (http://www.teenagecancertrust.org/).

### 5. What effect has European level funding (Framework Programme etc.) had on European paediatric oncology?

There is little evidence so far that European funding has had a measurable impact on resources for paediatric oncology.

### 6. Key areas to be addressed by the Commission in the next 5 years

Most clinical trials are European collaborative studies but very few provide funds which directly support participation of treatment centres in recruiting patients and collecting data. Nor do these studies provide resources for national data management support, for adequate travel and coordination costs of the trial leadership in the UK. The impact of the CTD has increased demands to improve standards of governance and resources for this increased activity have largely come from national funding streams. The coordinating role of SIOP Europe could be measurably enhanced by increased funds, not only for clinical trial network support but for education and multidisciplinary working. The outcome of the recent ENCCA project is yet to be determined.

## United Kingdom

**Table:** 

Name	David Walker
Country	UK
Position	Professor Paediatric Oncology
Institution	University of Nottingham

### 1. How is paediatric care and research delivered in the UK?

Twenty-two units in UK providing clinical care and integrated clinical trials programme. Centres vary in size and complexity form large units with full range of surgery, chemotherapy radiotherapy and bone marrow transplant to smaller units with some of these facilities. There is an associated large network of shared care centres, nationally approved and commissioned.

### 2. What are the key issues for paediatric oncology in delivering care and research in the UK?

In UK, we are undergoing a trials network reconfiguration of funding sources and organization. This is not complete and there are considerable uncertainties. The burden of GCP in implementing trials has downgraded trials network functionality as there was insufficient resource to meet burden of governance.

### 3. What are the sources of funding for your paediatric oncology research. What are your views on the balance between public and commercial sources of income?

Pre-clinical research in my field of neuro-oncology is largely dependent upon charity sourced funding. This has grown in past years linked to the lack of neuro-oncology research previously. Clinical trials funding has historically been strongly supported by major national charity CR-UK. Health services research is variously funded.

### 4. The state of patient information in the UK for childhood cancers

Well supported via a number of organizations CCLG, Cancer BACUP, CR-UK, Mac Millan.

### 5. What effect has European level funding (Framework Programme etc.) had on European paediatric oncology?

Until the recent SIOPE/ENCCA application Framework funding had had very limited impact. The recent EMEA review of unlicensed drugs for children was a welcome initiative.

### 6. Key areas to be addressed by the Commission in the next 5 years

Not specified.

## Figures and Tables

**Table 1:** Ten countries used for analysis in this study, with their ISO codes

**Code**	**Country**	**Code**	**Country**	**Code**	**Country**
CA	Canada	FR	France	SE	Sweden
CH	Switzerland	IT	Italy	UK	United Kingdom
DE	Germany	NL	Netherlands	US	United States
ES	Spain				

**Table 2:** Examples of research institutions (in USA and Canada) whose research outputs in paediatric oncology were sought by means of special search strings. For each institution, the main string (city and state/province) had to be present, plus either the zip or search string 2 (some institutions had additional search strings)

**Institution or city**	**Code**	**Main string**	**Zip**	**Search string 2**
Baptist Children’s Hospital	FLB	MIAMI, FL	33176	BAPTIST
Miami Children’s Hospital	FLM	MIAMI, FL	33155	MIAMI-CHILD
Children’s Hospital Oakland	CAO	OAKLAND, CA	94609	CHILD
Stanford University Medical Ctr	CAP	PALO-ALTO, CA	95798	STANFORD
Alberta Children’s Hospital	ABC	CALGARY, AB	T3B 6A8	CHILD
Kingston General Hospital	ONK	KINGSTON, ON	K7L 2V7	GEN-HOSP
Saskatoon Cancer Center	SKS	SASKATOON, SK	S7N 4H4	CANC

**Table 3:** Examples of some leading cities in European countries whose paediatric oncology research outputs were sought. The city name (and country) had to be present, plus one or more of the extra search strings (if more than one institution in the city). For some institutions, the presence of a ‘no’ string would nullify the match

**Country**	**City**	**Code**	**Search string 2**	**Search string 3**	**‘No’ strings**
France	LYON	FRB	BERARD		
France	PARIS	FRC	INST?CURIE		
France	PARIS	FRD	DEBRE		
Germany	DRESDEN	DEA			
Germany	BERLIN	DEB	HUMBOLDT	CHARITE	
Italy	BARI	ITA			
Italy	BOLOGNA	ITB	RIZZOLI		
Netherlands	AMSTERDAM	NLA	ACAD?MED	EMMA	FREE, VRIJE, VU-
Netherlands	AMSTERDAM	NLV	FREE	VRIJE	
UK	SUTTON	UKA			
UK	LONDON	UKE	LONDON?HOSP	BART	

**Table 4:** Examples of potential citation impact values (C_0–4_) for some journals commonly used for paediatric oncology papers, published in 2004 and cited 2004–8

***Journal***	***PCI***	***Journal***	***PCI***
*New England Journal of Medicine*	170.7	*European Journal of Cancer*	15.5
*Journal of Clinical Oncology*	40.8	*Bone Marrow Transplantation*	9.5
*Cancer Research*	33.0	*Journal of Pediatric Hematology Oncology*	6.7
*Clinical Cancer Research*	25.1	*Pediatric Radiology*	4.1
*Cancer*	18.9	*Pediatric Hematology and Oncology*	2.7

**Table 5:** Examples of research levels (RL_j_; 1 = clinical, 4 = basic) for some journals commonly used for paediatric oncology papers published in 2000–5

**Journal**	**RL**	**Journal**	**RL**
*Oncogene*	3.69	*European Journal of Cancer*	1.55
*Cancer Research*	3.32	*Journal of Pediatric Surgery*	1.40
*Blood*	2.94	*Pediatric Blood & Cancer*	1.24
*Leukemia*	2.42	*Journal of Pediatric Hematology Oncology*	1.15
*British Journal of Cancer*	1.84	*Pediatric Radiology*	1.04

**Table 6:** Sample sizes for the ten selected countries for the look-up of papers to determine funding acknowledgements in 1997–2000 and 2005–8

	***1997–2000***			***2005–8***	
**ISO**	**Output**	**Sample**	**ISO**	**Output**	**Sample**
US	3247	299	US	4182	289
DE	819	152	DE	1138	151
UK	756	607	UK	934	138
IT	553	126	IT	810	125
FR	592	126	FR	729	120
CA	415	105	CA	674	116
NL	304	89	NL	483	98
ES	221	79	ES	274	76
SE	193	73	SE	294	76
CH	127	60	CH	202	62
Total		1716	Total		1251

Note: UK sample for 1997–2000 would have been only 142 but 607 papers had been looked up already. CH sample would have been 58 but was increased to the minimum of 60.

**Table 7:** List of codes for sectors and sub-sectors for the funders of paediatric oncology research

**Sector and sub-sector**	**Code**	**Sector and sub-sector**	**Code**
Government		Industry	
Government department	GD	Biotechnology company	BT
Government agency	GA	Industry, non-pharma	IN
Local or regional authority	LA	Industry, pharma	IP
Private-non-profit		Subsidiary, non-pharma	SN
Charity (collecting)	CH	Subsidiary, pharma	SP
Foundation (endowed)	FO		
Hospital trustees	HT	International organization	*XN*
Mixed (academic own)	MI	European organization	*EU*
Non-profit (other)	NP	(these are used as country codes)	

Note: Other non-profit funders are PNP bodies not primarily supporting research, e.g. professional associations, trade groupings. LA denotes US states, Canadian provinces, German Länder, etc.

**Table 8:** Annual outputs of paediatric oncology papers (articles and reviews, fractional counts) from selected countries and the other 20 EU Member States, 1997–2008

	**1997–2000**		**2001–4**		**2005–8**	
**World**	**2296**	**%**	**2440**	**%**	**3051**	**%**
US	739	32.2	750	30.8	921	30.2
DE	172	7.5	193	7.9	215	7.0
EU20	138	6.0	162	6.6	206	6.7
UK	152	6.6	157	6.4	167	5.5
IT	116	5.1	129	5.3	166	5.4
FR	126	5.5	120	4.9	143	4.7
CA	76	3.3	78	3.2	118	3.9
NL	57	2.5	51	2.1	82	2.7
ES	50	2.2	42	1.7	53	1.7
SE	38	1.6	40	1.6	48	1.6
CH	21	0.9	24	1.0	27	0.9

**Table 9:** North American institutions publishing at least 100 papers (fractional count) in paediatric oncology, 1997–2008

**Code**	**Institution**	**1997–2000**	**2001–4**	**2005–8**	**Total**
TNM	St Jude Children’s Hospital, Memphis, TN	163.8	177.5	222.8	564
PAC	Children’s Hospital of Philadelphia, Philadelphia, PA	101.0	114.7	146.5	362
MAD	Dana-Farber Cancer Institute and Children’s Hospital, Boston, MA	81.0	107.3	139.0	327
ONT	Hospital for Sick Children, Toronto, ON, Canada	72.9	91.2	131.7	296
TXH	M.D. Anderson Cancer Center, Houston, TX	79.3	104.1	107.3	291
NYL	Memorial Sloan Kettering, New York, NY	77.6	88.2	80.1	246
MDB	National Cancer Institute, Bethesda, MD	72.2	82.9	87.3	242
MDJ	Johns Hopkins Hospital, Baltimore, MD	44.5	53.9	68.2	166
MNU	University of Minnesota Cancer Center, Minneapolis, MN	42.8	54.2	65.2	162
CAF	UCSF School of Medicine, San Francisco, CA	46.6	42.1	50.9	140
MNR	Mayo Clinic, Rochester, MN	36.2	43.2	51.9	131
PAG	University of Pittsburgh, Pittsburgh, PA	46.6	33.3	46.9	127
TXB	Texas Children Cancer Control, Baylor College of Medicine, Houston, TX	27.1	34.8	58.5	120
MIA	C.S. Mott Children’s Hospital, Ann Arbor, MI	23.2	37.5	46.8	108
NCD	Duke University Medical Center, Durham, NC	28.6	36.1	40.2	105
NYP	Columbia Presbyterian College of Physicians & Surgeons, New York, NY	28.2	34.7	41.5	104
MID	Children’s Hospital of Michigan, Detroit, MI	33.9	32.5	35.2	102

**Table 10:** European cities, universities and institutes publishing at least 100 papers (fractional count) in paediatric oncology, 1997–2008

**Code**	**Institution**	**1997–2000**	**2001–4**	**2005–8**	**Total**
UKU	University College London, England	66.0	77.2	88.8	232
NLA	University of Amsterdam, Netherlands	65.3	56.1	70.4	192
ESM	Madrid (city), Spain	63.1	44.8	54.6	162
NLR	Erasmus University, Rotterdam, Netherlands	39.8	42.0	76.1	158
SES	Karolinska Institutet, Stockholm, Sweden	35.6	57.7	64.4	158
ITM	University of Milan, Italy	51.8	41.4	58.6	152
DES	University of Munster, Germany	43.8	52.5	52.3	149
FRV	Institut Gustave Roussy, Villejuif, France	45.2	45.8	52.3	143
UKM	Manchester (city), England	47.7	42.7	47.4	138
DEM	München (city), Germany	46.7	41.7	44.1	133
DED	Düsseldorf (city), Germany	43.0	45.2	40.5	129
DEH	Heidelberg (city), Germany	34.6	32.1	61.1	128
ITP	Padova (city), Italy	26.1	38.7	56.3	121
DEV	Hannover (city), Germany	44.1	30.9	44.9	120
UKB	Nottingham (city), England	37.7	34.5	45.9	118
ITG	Istituto Gaslini, Genova, Italy	20.4	42.9	52.6	116
ESB	Barcelona (city), Spain	43.0	31.9	39.0	114
UKN	Newcastle-upon-Tyne (city), England	34.4	40.3	38.6	113
ITR	Univ. La Sapienza (Bambino Gesù), Rome, Italy	29.8	40.2	43.0	113
DEB	Humboldt Univ, (Charité), Berlin, Germany	25.7	33.5	51.2	110

**Table 11:** Leading North American research institutions in paediatric oncology, 1997–2008, with three measures of ‘quality’—mean percentage of reviews, potential citation impact (PCI) and actual citation impact (ACI) (1997–2004 papers, 5-year citation window); all relative to world mean values of 6.17%, 11.04 cites and 8.62 cites. For colour coding, see text above

**Code**	**Institution**	**% rev/W**	**PCI/W**	**ACI/W**
MAD	Dana-Farber Cancer Institute and Children Hospital, Boston	1.88	2.02	2.76
MDB	National Cancer Institute, Bethesda	1.71	1.94	2.45
NYP	Columbia Presbyterian College of Physicians & Surgeons, New York	1.97	1.48	2.53
MNU	University of Minnesota Cancer Center, Minneapolis	1.64	1.80	2.23
CAF	UCSF School of Medicine, San Francisco	1.67	1.67	2.18
MNR	Mayo Clinic, Rochester	1.31	1.66	2.27
TXH	M.D. Anderson Cancer Control, Houston	1.57	1.59	1.96
MDJ	Johns Hopkins Hospital, Baltimore	1.66	1.58	1.80
PAC	Children’s Hospital, Philadelphia	1.46	1.60	1.91
TNM	St Jude Children’s Hospital, Memphis	1.26	1.68	1.94
NYL	Memorial Sloan Kettering, New York	1.18	1.75	1.94
ONT	Hospital for Sick Children, Toronto	1.93	1.16	1.16
MID	Children’s Hospital of Michigan, Detroit	1.10	1.30	1.84
MIA	C.S. Mott Children’s Hospital, Ann Arbor	1.02	1.48	1.67
TXB	Texas Children Cancer Control, Baylor College of Medicine, Houston	1.06	1.44	1.59
PAG	University of Pittsburgh, Pittsburgh	1.38	1.30	1.32
NCD	Duke University Medical Center, Durham	1.21	1.36	1.43

**Table 12:** Leading European cities and universities in paediatric oncology, 1997–2008, with three measures of ‘quality’—mean percentage of reviews, potential citation impact (PCI) and actual citation impact (ACI) (1997–2004 papers, 5-year citation window); all relative to world mean values of 6.17%, 11.04 cites and 8.62 cites. For colour coding, see text above Table 11

**Code**	**Institution**	**% rev/W**	**PCI/W**	**ACI/W**
NLA	University of Amsterdam, Netherlands	2.42	1.37	1.74
NLR	Erasmus University, Rotterdam, Netherlands	1.21	1.85	2.18
DES	University of Munster, Germany	2.13	1.17	1.71
FRV	Institut Gustave Roussy, Villejuif, France	1.78	1.37	1.51
UKU	University College London, England	1.39	1.35	1.81
UKN	Newcastle-upon-Tyne (city), England	1.29	1.67	1.57
UKM	Manchester (city), England	1.14	1.46	1.58
DEH	Heidelberg (city), Germany	0.89	1.39	1.69
DEB	Humboldt Univ. (Charité), Berlin, Germany	0.57	1.56	1.71
UKB	Nottingham (city), England	0.97	1.53	1.32
DEV	Hannover (city), Germany	0.45	1.49	1.86
ITG	Istituto Gaslini, Genova, Italy	0.91	1.30	1.55
SES	Karolinska Institute, Stockholm, Sweden	0.51	1.52	1.69
ITM	University of Milan, Italy	1.20	1.24	1.26
ITR	Univ. La Sapienza (Bambino Gesù), Rome, Italy	0.89	1.34	1.32
DED	Düsseldorf (city), Germany	1.10	1.03	1.26
ITP	Padova (city), Italy	0.51	1.36	1.29
ESB	Barcelona (city), Spain	0.78	1.14	1.13
DEM	München (city), Germany	0.55	1.04	1.29
ESM	Madrid (city), Spain	0.72	0.92	0.70

**Table 13:** Foreign contributions to paediatric oncology papers from selected countries, EU20 and the Rest of the World (RoW), 1997–2008, fractional counts of articles + reviews

**H\G**	**US**	**DE**	**EU20**	**UK**	**IT**	**FR**	**CA**	**NL**	**ES**	**SE**	**CH**	**RoW**
US		135	137	95	82	58	166	55	17	40	24	358
DE	130		111	58	41	35	16	60	6	16	33	65
EU20	146	155		71	46	70	17	60	9	48	15	60
UK	127	51	71		53	57	29	35	11	21	10	103
IT	89	30	43	46		32	9	15	10	10	9	41
FR	65	32	42	43	39		12	18	10	17	13	57
CA	305	21	14	30	10	16		6	2	4	5	57
NL	64	56	59	41	20	24	6		5	10	5	30
ES	24	8	15	12	13	12	2	9		3	2	13
SE	41	18	51	19	12	10	6	14	3		1	46
CH	36	59	21	14	12	29	5	5	1	1		18

**Table 14:** Ratio of observed to expected foreign contributions to each of the selected countries’ (listed in column 1) paediatric oncology research papers; fractional count basis, 1997–2008. For colour coding, see text on previous page

**Ratio**	**US**	**DE**	**EU20**	**UK**	**IT**	**FR**	**CA**	**NL**	**ES**	**SE**	**CH**	**RoW**
US		1.07	1.25	0.92	0.92	0.69	2.81	1.34	0.54	1.47	1.56	0.75
DE	0.68		2.77	1.54	1.26	1.14	0.74	4.00	0.52	1.61	5.87	0.37
EU20	0.63	2.79		1.56	1.17	1.88	0.65	3.31	0.65	4.00	2.21	0.28
UK	0.68	1.13	1.81		1.66	1.89	1.37	2.38	0.98	2.16	1.81	0.60
IT	0.82	1.14	1.88	2.14		1.82	0.73	1.75	1.52	1.76	2.80	0.41
FR	0.57	1.17	1.77	1.92	2.02		0.94	2.02	1.47	2.89	3.89	0.55
CA	2.02	0.58	0.44	1.01	0.39	0.66		0.51	0.22	0.51	1.13	0.41
NL	0.63	2.29	2.77	2.05	1.16	1.47	0.52		0.82	1.90	1.67	0.32
ES	0.67	0.93	2.01	1.71	2.14	2.09	0.50	3.21		1.62	1.90	0.40
SE	0.59	1.08	3.50	1.38	1.01	0.89	0.76	2.56	0.72		0.49	0.72
CH	0.57	3.91	1.59	1.13	1.12	2.86	0.71	1.01	0.26	0.31		0.31

**Table 15:** Statistical significance (percent) between observed and expected numbers of papers from a given foreign country (column) within the papers published by one of the selected countries (row). For shading codes see previous page; n.s. = not significant at 5% level

**Significant**	**US**	**DE**	**EU20**	**UK**	**IT**	**FR**	**CA**	**NL**	**ES**	**SE**	**CH**	**RoW**
US		n.s.	0.69	n.s.	n.s.	0.32	0.00	2.67	1.10	1.16	2.00	0.00
DE	0.00		0.00	0.07	n.s.	n.s.	n.s.	0.00	n.s.	n.s.	0.00	0.00
EU20	0.00	0.00		0.01	n.s.	0.00	n.s.	0.00	n.s.	0.00	0.23	0.00
UK	0.00	n.s.	0.00		0.01	0.00	n.s.	0.00	n.s.	0.04	n.s.	0.00
IT	2.02	n.s.	0.00	0.00		0.06	n.s.	4.35	n.s.	n.s.	0.04	0.00
FR	0.00	n.s.	0.01	0.00	0.00		n.s.	0.23	n.s.	0.00	0.00	0.00
CA	0.00	0.94	0.09	n.s.	0.12	n.s.		n.s.	1.90	n.s.	n.s.	0.00
NL	0.00	0.00	0.00	0.00	n.s.	4.11	n.s.		n.s.	2.49	n.s.	0.00
ES	1.58	n.s.	0.17	n.s.	0.32	1.21	n.s.	0.04		n.s.	n.s.	0.00
SE	0.00	n.s.	0.00	n.s.	n.s.	n.s.	n.s.	0.00	n.s.		n.s.	0.77
CH	0.99	0.00	2.24	n.s.	n.s.	0.00	n.s.	n.s.	n.s.	n.s.		0.00

**Table 16:** Matrix of increases or decreases in the amount of inter-country collaboration in paediatric oncology research, from 1997–2000 to 2005–8. Colour coding: bright green, a definite increase; light green, a probable increase; light yellow, a probable decrease; pink, a definite decrease

**Host/guest**	**US**	**DE**	**EU20**	**UK**	**IT**	**FR**	**CA**	**NL**	**ES**	**SE**	**CH**	**RoW**
US												
DE												
EU20												
UK												
IT												
FR												
CA												
NL												
ES												
SE												
CH												

**Table 17:** Propensity for researchers in the leading US and Canadian paediatric oncology institutions (for codes, see Table 9) to collaborate with researchers from the nine selected foreign countries, the EU20 and the Rest of the World (RoW), 1997–2008. For colour coding, see text after Table 13

	**CA**	**CH**	**DE**	**ES**	**FR**	**IT**	**NL**	**SE**	**UK**	**US**	**EU20**	**RoW**
ONT		0.74	0.33	0.29	0.18	0.16	0.51	0.64	1.28	2.22	0.34	0.43
CAF	4.93	4.84	1.09	0.88	3.68	0.88	1.24	0.58	1.43		1.52	0.79
MAD	3.87	2.33	0.68	0.26	0.75	1.56	2.44	6.99	1.47		3.29	0.64
MDB	2.59	0.93	2.15	0.06	0.78	1.29	1.82	2.47	1.21		1.74	1.25
MDJ	3.86	3.20	1.40	0.16	1.25	0.69	7.07	4.32	0.81		1.11	0.92
MIA	3.04	9.73	2.40	0.00	1.51	1.04	2.10	0.48	1.38		2.11	0.74
MID	7.61	5.97	1.17	0.00	0.09	0.08	4.88	1.64	0.76		0.25	1.32
MNR	4.50	3.74	0.61	0.20	1.04	0.62	0.73	3.76	3.63		1.34	1.00
MNU	9.35	0.00	2.26	0.53	2.13	0.66	0.84	0.65	2.13		0.27	0.59
NCD	9.59	9.30	1.42	0.89	0.51	0.38	2.33	0.00	0.25		0.31	1.12
NYL	6.04	1.23	0.80	5.50	0.98	0.21	1.15	1.45	0.99		1.29	1.25
NYP	3.42	0.00	0.00	0.33	1.31	2.25	1.58	3.39	1.48		1.14	1.52
PAC	8.49	6.06	0.87	0.54	0.13	1.07	0.67	1.64	1.30		1.51	0.98
PAG	7.13	0.57	1.58	3.64	1.95	0.35	0.21	0.00	0.61		2.95	0.72
TNM	5.46	1.67	2.00	0.03	0.99	0.71	1.86	0.50	1.88		0.33	1.24
TXB	13.28	8.82	0.24	0.00	1.06	0.83	2.00	0.00	0.29		1.57	0.59
TXH	3.75	2.33	2.78	0.75	0.80	0.90	1.86	0.33	0.77		1.59	1.16

**Table 18:** Propensity for researchers in the leading European paediatric oncology cities and universities (for codes, see Table 10) to collaborate with researchers from the nine selected foreign countries, the EU20 and the Rest of the World (RoW), 1997–2008. For colour coding, see text after Table 13

	**CA**	**CH**	**DE**	**ES**	**FR**	**IT**	**NL**	**SE**	**UK**	**US**	**EU20**	**RoW**
DEB	0.36	6.01		0.07	1.93	1.14	4.92	1.32	0.74	0.75	4.12	0.29
DED	0.69	4.73		0.25	0.57	0.91	4.23	0.50	3.18	0.90	3.01	0.22
DEH	0.42	5.02		1.40	1.58	1.18	2.58	4.00	1.91	0.68	2.14	0.61
DEM	0.43	8.72		0.37	1.79	1.48	1.84	2.14	2.34	0.62	2.65	0.49
DES	1.80	9.57		0.17	0.75	0.46	7.48	0.57	1.42	0.54	4.56	0.12
DEV	1.54	6.75		0.00	0.58	1.26	2.99	1.28	0.98	0.79	4.22	0.38
ESB	0.60	1.01	0.90		2.83	2.46	2.84	0.50	2.04	0.75	1.76	0.27
ESM	0.51	0.41	1.01		0.57	1.31	4.21	2.43	1.91	1.01	1.27	0.50
FRV	1.08	3.67	1.29	0.75		2.24	4.73	1.91	3.87	0.39	1.27	0.37
ITG	1.54	1.76	1.75	1.02	2.60		1.68	1.49	2.07	0.66	1.59	0.51
ITM	1.15	3.70	1.97	1.93	1.28		2.99	0.27	2.24	0.67	1.54	0.53
ITP	0.50	4.72	2.52	1.93	2.27		3.90	0.41	2.90	0.44	1.92	0.19
ITR	0.33	1.32	1.09	0.90	3.06		1.79	0.71	2.10	0.99	1.29	0.51
NLA	0.59	1.37	2.37	0.50	2.27	1.08		2.29	1.67	0.68	2.13	0.40
NLR	0.39	0.63	2.93	0.66	1.32	0.97		1.60	1.83	0.64	3.55	0.20
SES	0.54	0.36	0.65	0.81	0.66	0.93	2.04		1.03	0.81	3.33	0.87
UKB	1.86	2.67	1.08	1.02	4.16	1.07	3.20	2.70		0.48	1.60	0.59
UKM	1.56	0.47	1.04	0.24	1.08	0.86	3.08	3.92		1.09	1.68	0.59
UKN	1.12	0.62	1.76	0.44	2.49	2.44	3.94	3.02		0.43	2.63	0.38
UKU	1.71	2.92	0.61	0.82	1.07	2.03	3.31	1.90		0.83	1.74	0.71

**Table 19:** Percentages of paediatric oncology papers receiving support from different funding sectors in two quadrennia: 1997–2000 (1) and 2005–8 (3) from ten selected countries. GOV = national government (including regions), PNP = national private-non-profit, INDY = industry (from any country), INTL = international, OTHER = foreign government and PNP

**ISO**	**Quadr.**	**Papers**	**GOV %**	**PNP %**	**INDY %**	**INTL %**	**OTH %**	**NONE %**
CA	1	92	21.7	25.0	8.7	0.0	26.1	45.7
	3	95	24.2	14.7	7.4	0.0	25.3	46.3
CH	1	52	9.6	19.2	11.5	0.0	32.7	51.9
	3	52	9.6	17.3	7.7	3.8	42.3	44.2
DE	1	131	16.8	34.4	4.6	1.5	9.9	47.3
	3	119	10.1	22.7	3.4	3.4	21.8	56.3
ES	1	64	31.3	12.5	3.1	4.7	10.9	59.4
	3	65	29.2	13.8	1.5	4.6	24.6	49.2
FR	1	87	9.2	36.8	4.6	1.1	17.2	49.4
	3	100	23.0	36.0	3.0	8.0	25.0	44.0
IT	1	106	42.5	36.8	2.8	0.9	12.3	36.8
	3	103	27.2	35.9	1.0	5.8	14.6	48.5
NL	1	77	5.2	31.2	5.2	1.3	9.1	57.1
	3	91	3.3	25.3	2.2	4.4	22.0	52.7
SE	1	59	40.7	66.1	6.8	5.1	25.4	16.9
	3	69	33.3	59.4	0.0	10.1	39.1	18.8
UK	1	607	18.1	39.7	9.1	5.4	23.2	41.5
	3	104	10.6	23.1	1.9	12.5	23.1	51.0
US	1	252	40.9	32.5	4.8	0.0	10.7	40.5
	3	250	40.8	28.0	7.6	0.0	8.4	42.4

**Table 20:** Numbers and percentages of paediatric oncology papers acknowledging sub-sectors of support in the ten selected countries, 1997–2000 and 2005–8 together. LA = local or regional authority, CH = collecting charity, FO = endowed foundation, BT = biotech company, EU = European Union

	**Papers**	**LA**	**LA %**	**CH**	**CH %**	**FO**	**FO %**	**BT**	**BT %**	**EU**	**EU %**
CA	187	5	2.7	23	12.3	7	3.7	2	1.1	0	0.0
CH	105	0	0.0	13	12.4	6	5.7	0	0.0	0	0.0
DE	250	2	0.8	45	18.0	15	6.0	0	0.0	7	2.8
ES	129	7	5.4	2	1.6	6	4.7	0	0.0	6	4.7
FR	187	3	1.6	32	17.1	28	15.0	1	0.5	8	4.3
IT	209	5	2.4	51	24.4	19	9.1	0	0.0	7	3.3
NL	168	0	0.0	37	22.0	8	4.8	0	0.0	5	3.0
SE	128	7	5.5	52	40.6	54	42.2	0	0.0	9	7.0
UK	711	0	0.0	225	31.6	52	7.3	9	1.3	40	5.6
US	502	11	2.2	90	17.9	35	7.0	7	1.4	0	0.0

**Table 21:** Leading funders of paediatric oncology research in ten selected countries, 1997–2000 and 2005–8

Canada	*N*	%
Canadian Institutes of Health Research	31	16.6
National Cancer Institute of Canada	10	5.3
Canadian Cancer Society	9	4.8
Miscellaneous Canadian foundations	7	3.7
Natural Sciences and Engineering Research Council	5	2.7
Heart and Stroke Foundation of Canada	4	2.1
*Switzerland*	*N*	*%*
Swiss National Science Foundation	9	8.7
Swiss Cancer League	9	8.7
Miscellaneous Swiss Foundations	3	2.9
Miscellaneous Swiss Charities	3	2.9
University of Zurich own funds	3	2.9
*Germany*	*N*	*%*
German Cancer Society	34	13.6
Deutsche Forschung Gesell.	20	8
Miscellaneous German charities	13	5.2
Miscellaneous German non-profits	9	3.6
Ministry of Educ & Science	7	2.8
Miscellaneous German universities	7	2.8
European Commission	6	2.4
Deutsche Krebsforsch. Zent.	6	2.4
Miscellaneous German foundations	6	2.4
*Spain*	*N*	*%*
Fondo de Investig. Sanitarias	25	19.4
European Commission	6	4.7
Caja de Navarra	5	3.9
Miscellaneous Spanish non-profits	5	3.9
Ministerio de Educación y Ciencia	4	3.1
Com. Inter. Inv. Cien. y Tech	3	2.3
Instituto de Salud Carlos III	3	2.3
Jose Carreras foundation	3	2.3
*France*	*N*	*%*
Miscellaneous French foundations	23	12.3
INSERM	18	9.6
Fondation de France	14	7.5
Ligue Nat Franc contre le Canc	14	7.5
Miscellaneous French non-profits	10	5.3
European Commission	8	4.3
Institut Gustave Roussy	8	4.3
Ligue Region. contre le Canc.	8	4.3
Ministère de Santé	6	3.2
Institut Curie	5	2.7
Intl Agency for Res on Cancer	4	2.1
Fondation pr la Rech Medicale	4	2.1
Miscellaneous French charities	4	2.1
*Italy*	*N*	*%*
Assoc Italiana Ricerc sul Canc	53	25.3
Minist. Educ & Research	36	17.2
Consiglio Naz delle Ricerche	33	15.8
Miscellaneous Italian foundations	17	8.1
Miscellaneous Italian non-profits	14	6.7
Ministry of Health	9	4.3
Miscellaneous Italian charities	8	3.8
European Commission	7	3.3
Compagnia di San Paolo	7	3.3
Istituti Scientifici Rizzoli	6	2.9
Com M L Verga Leucem Infant	5	2.4
I R C C S	5	2.4
Miscellaneous Italian universities	5	2.4
*Netherlands*	*N*	*%*
Netherlands Cancer Society	33	19.6
Miscellaneous Dutch foundations	7	4.2
Miscellaneous Dutch universities	5	3.0
European Commission	4	2.4
Netherlands Organisation for Scientific Research	4	2.4
*Sweden*	*N*	*%*
Children’s Cancer Foundation	41	32.0
Swedish Cancer Society	37	28.9
Miscellaneous Swedish foundations	23	18.0
Sweden Council for Medical Research	18	14.1
Research Council for Engineering Sciences	17	13.3
King Gustav V Jubilee fund	17	13.3
European Commission	9	7.0
Assar Gabrielsson foundation	5	3.9
Stockholm County Council	5	3.9
Swedish Society of Medicine	5	3.9
University of Lund funds	4	3.1
Miscellaneous Swedish charities	4	3.1
Sweden National Institute of Radiation Protection	3	2.3
Miscellaneous Swedish universities	3	2.3
Miscellaneous Swedish non-profits	3	2.3
*United Kingdom*	*N*	*%*
Cancer Research UK^§^	140	19.7
Medical Research Council	65	9.1
Leukaemia Research Fund	62	8.7
European Commission	40	5.6
Department of Health	38	5.3
Sainsbury Family trusts	23	3.2
North of England Cancer Research Fund	22	3.1
Wellcome Trust	14	2.0
*United States*	*N*	*%*
National Institutes of Health*	139	27.7
National Cancer Institute	91	18.1
American Lebanese Syrian Associated Charities	37	7.4
Miscellaneous US charities	31	6.2
Miscellaneous US foundations	26	5.2
American Cancer Society	14	2.8
Miscellaneous US non-profits	12	2.4
Department of Defense	11	2.2

This total includes all other of the institutes within the NIH except the NCI.

§ This total includes papers acknowledging the Cancer Research Campaign and the Imperial Cancer Research Fund, which merged to form Cancer Research UK.

**Table 22:** List of 21 leading pharmaceutical companies with countries, names and search terms used to identify their papers in paediatric oncology (P) and overall (WoS), numbers of paediatric oncology papers overall and of research papers annually in 1997–2008 and the percentage of the former in the latter

**Company**	**ISO**	**Search terms**	**WoS**	**P**	**%**
GlaxoSmithKline	UK	Glaxo, SmithKline	1089	27	0.21
Pfizer	US	Agouron, Parke-Davis, Pfizer, Pharmacia, Searle, Sugen, Upjohn, Warner-Lambert	1050	22	0.17
Merck Inc	US	Dohme, MSD, Merck NOT Merck-KgaA	871	11	0.11
Novartis	CH	Chiron, CIBA-Geigy, Novartis, Sandoz	860	35	0.34
AstraZeneca	UK	Astra, Zeneca	672	17	0.21
Eli Lilly	US	Lilly	548	9	0.14
Hoffman La Roche	CH	Chugai, Genentech, F-Hoffmann	439	12	0.23
Johnson & Johnson	US	Alza, Centocor, Cilag, Ethicon, Johnson-&-Johnson, McNeil, Ortho, Scios	400	19	0.40
Aventis	FR	Aventis, L’Oreal, Sanofi, Synthelabo	385	15	0.32
Bristol-Myers Squibb	US	Bristol-Myers, Squibb	327	7	0.18
Amgen	US	Abgenix, Alantos, Amgen, Avidia, Ilypsa, Immunex, Kinetix, Tularik	306	19	0.52
Wyeth	US	American-Home-Products, Ayerst, Wyeth	291	6	0.17
Schering-Plough	US	Schering NOT Schering-AG	267	9	0.28
Sankyo	JP	Daiichi, Sankyo	210	3	0.12
Novo Nordisk	DK	Novo-Nordisk	187	3	0.13
Boehringer Ingel’m	DE	Boehringer AND Ingelheim	186	6	0.27
Astellas	JP	Astellas, Fujisawa-Pharm, Yamanouchi-Ph.	167	7	0.35
Schering KgaA	DE	Schering-KgaA	101	2	0.17
Takeda Chemical	JP	Takeda NOT Takeda-Hosp	88	1	0.10
Eisai	JP	Eisai	72	2	0.23
Merck AG	DE	Merck-KgaA	59	0	0.00

**Table 23:** Estimated funding for paediatric oncology research in 2008, US dollars (million)

Sector	Spend, $ m	Per cent
Public, incl. international	656	53
Private-non-profit	328	27
Commercial	245	20

## Footnotes

1 For 2004 papers, the values for each journal were obtained by division of the numbers of citations to articles and reviews only by the number of such papers; for earlier years the numerator also included a small number of citations to other document types. This discrepancy has led to some criticism in the literature.

2 These are usually slightly above the nominal values as the actual number of world papers with the given number of citations normally exceeds the required number.

3 Care was taken to avoid titles with words such as “endometrial”, “atrial” and “terrestrial”.

4 The figure is lower because very little cancer research is carried out in low-income countries.

## Annex A: filters to select papers on the WoS

### A.1 Oncology subfield

SO=(A*ONCOL* OR A*CANCER* OR B*CANCER* OR B*TUMOR* OR C*CANCER* OR CANCER OR CANCER-* OR CL*LYMPHOMA* OR C*ONCOL* OR CARCINOGENESIS OR CHEMOTHERAP* OR CLIN*METASTAS* OR E*CANCER* OR E*CARCINOG* OR E*ONCOL* OR F*CANCER* OR G*CANCER* OR G*ONCOL* OR H*ONCOL* OR INT*CANCER* OR INT*ONCOL* OR INVAS*METAST* OR INVEST*NEW*DRUG* OR J*CANCER* OR J*CHEMOTHERAPY OR J*ONCOL* OR J*IMMUNOTHER* OR L*CANCER* OR LEUK*RES* OR LEUKEMIA* OR L*ONCOL*)

SO=(MOL*CANCER* OR MED*ONCOL* OR MELANOMA* OR MOL*CARCINO* OR N*CANCER* OR N*ONCOL* OR NEOPLASIA OR NEOPLASM* OR ONCOGENE* OR ONCOL* OR O*ONCOL* OR ONKOLOG* OR P*CANCER* OR P*HEMATOL* OR P*ONCOL* OR P*TUMOR* OR RADIAT*RES* OR R*ONCOL* OR S*CANCER* OR S*ONCOL* OR STRAHL*ONKOL* OR T*CANCER* OR TERATOGEN*CARCINO* OR TUMOR* OR U*ONCOL* OR VOP*ONKOL* OR WSPOL*ONKOL*)

TI=(ADENOMA* OR AML OR ADENOCARCINOMA* OR ADRIAMYCIN* OR ANASTROZOLE OR ANGIOSARCOMA* OR ANTICANCER* OR ANTICARCINO* OR ANTINEOPLAS* OR ANTIPROLIF* OR (ANTITUMOR* NOT NECROSIS) OR ARIMIDEX* OR AROMATASE OR ASTROCYTOMA* OR BCL2 OR BCL6 OR BCR ABL OR BCR/ABL OR BEVACIZUMAB OR BICALUTAMIDE OR BIOREDUCTIVE OR BRCA1 OR BRCA2 OR BLEOMYCIN OR BRAF OR BRYOSTATIN* OR BUSULFAN OR CADHERIN* OR CAELYX* OR CANCER* OR CAPECITABINE OR CARBOGEN OR CARBOPLATIN OR CARMUSTINE OR CARCINO* OR CDKN2A OR CDX2 OR CEA OR CERVICAL SMEAR* OR CHEMOPREVENT* OR CHEMOSENSITIV* OR CHEMOTHERAP* OR CHLORAMBUCIL OR CHORIOCARCINOMA* OR CIN OR CISPLATIN)

TI=(CLL OR CML OR COMBRETASTATIN* OR CRANIOPHARYNGIOMA* OR CYCLOPHOSPHAMIDE OR CYTARABINE OR CYTOSINE ARABINOSIDE OR DACARBAZINE OR DAUNORUBICIN OR DOCETAXEL OR DOXORUBICIN* OR EBV OR EORTC OR EPENDYMOMA* OR EPIRUBICIN* OR EPSTEIN BARR OR ERBB* OR ERLOTINIB OR ESTRAMUSTINE OR ETOPOSIDE* OR ETV6 OR EXEMESTANE OR FIBROMA* OR FIBROSARCOMA* OR FLI1 OR FLUOROURACIL* OR FOS OR GANGLIOGLIOMA* OR GANGLIONEUROBLASTOMA* OR GEFITINIB OR GEMCITABINE* OR GERMINOMA* OR GLIOBLASTOMA* OR GLIOMA* OR GLEVEC* OR GLIVEC OR HEMANGIOBLASTOMA* OR HEMANGIOENDOTHELIOMA* OR HEPATOBLASTOMA OR HEPATOCARCINOGEN* OR HEPATOMA* OR HER2 OR HERCEPTIN* OR HODGKIN DISEASE OR HODGKINS OR (HPV* NOT PARVO*))

TI=(HRPT2 OR HYPERNEPHROMA* OR IDARUBICIN OR IFOS*AMIDE* OR IMATINIB OR INSULINOMA* OR INTRATUMOR* OR IRESSA* OR IRINOTECAN OR (IRRADIATION AND FRACTIONATED) OR JUN OR LETROZOLE OR LEUKEM* OR LI FRAUMENI OR LYMPHOMA* OR MALIGNANC* OR MALIGNANT OR MAMMOGRA* OR MASTECTOM* OR MEDULLOBLASTOMA* OR MELANOMA* OR MELPHALAN* OR MENINGIOMA* OR MESOTHELIOMA* OR METASTAS* OR METASTAT* OR METHYLGUANINE OR MLH1 OR MLL OR MSH OR MSH2 OR MUC1 OR MYB OR (MYC NOT (C OR N)) OR MYELODYSPLAS* OR MYELOMA* OR NEOPLAS* OR NEPHROBLASTOMA* OR NEPHROMA* OR NEURINOMA* OR NEUROBLASTOMA* OR NEUROMA*)

TI=(NF1 OR NF2 OR NHL OR NUP98 OR OLIGODENDROGLIOMA* OR ONCOGEN* OR ONCOLOG* OR ONCOPROTEIN* OR OSTEOSARCOMA* OR OXALIPLATIN OR P16INK4A OR P53 OR PACLITAXEL* OR PAP SMEAR* OR PAPILLOMA* OR PAX3 OR PEUTZ OR PHEOCHROMOCYTOMA* OR PHTOTODYNAMIC THERAP* OR PML/RAR* OR PREDNISOLONE OR (PML AND (APOPTO* OR GENE OR NUCLEAR OR NUCLEUS OR PROTEIN* OR RAR* OR UBIQUITIN*)) OR PMS1 OR POLYCYTHEMIA RUBRA OR PROTOONCOGEN* OR PSA OR PTEN OR RAD51 OR RADIOSENSI* OR RADIOTHERAP* OR RALTITREXED OR RAS OR RB1 OR RET OR RETINOBLASTOMA* OR RHABDOMYOSARCOMA* OR SARCOMA* OR SCHWANNOMA* OR SEMINOMA*) TI=(SRC OR STREPTOZOCIN OR TAMOXIFEN OR TARCEVA* OR TAXOL OR TAXOTERE* OR TEMOZOL*MIDE OR TEMODAL OR TERATOMA* OR THIOTEPA OR THYMOMA* OR TOMUDEX* OR (TOPOISOMERASE AND INHIBITOR) OR TOPOTECAN OR TP53 OR TRASTUZUMAB OR TREOSULFAN* OR TSC1 OR (TUMOR* NOT NECROSIS) OR (TRANSPLANT* AND (MARROW OR STEM CELL*)) OR VINBLASTINE OR VINCRISTINE OR VINORELBINE OR WALDENSTROM* OR XERODERMA PIGMENTOSUM OR ZOLEDRONIC ACID)

### A.2 Paediatrics subfield

SO=(A*CHILD* OR A*PAEDIAT* OR A*PEDIAT* OR B*NEONAT* OR B*PEDIAT* OR C*PEDIAT* OR C*PERINAT* OR CHILD* OR D*CHILD* OR DEVELOP*PHYSIOL* OR E*PAEDIAT* OR E*PEDIAT* OR EARLY-HUMAN* OR F*PEDIAT* OR H*PAEDIAT* OR I*PAEDIAT* OR I*PEDIAT* OR J*CHILD* OR J*PAEDIAT* OR J*PEDIAT* OR J*PERINAT* OR K*PADIAT* OR M*CHILD* OR M*PEDIAT* OR N*PEDIAT* OR PAEDIAT* OR PEDIAT* OR S*PEDIAT* OR S*PERINAT* OR T*PEDIAT* OR W*PEDIAT*)

TI=(ACUTE LYMPHOBLASTIC OR ADOLESCEN* OR ANTENATAL OR ATRESIA OR BIRTH OR BIRTHS OR BOY OR BOYS OR BREAST MILK OR BREAST FED OR (CEREBRAL PALSY NOT ADULT*) OR CHILD OR CHILDHOOD OR CHILDREN* OR CHORIOAMNIONITIS OR (COARCTATION NOT ADULT*) OR CONGENITAL OR DOWN SYNDROME OR DOWNS SYNDROME OR DUCTUS ARTERIOSUS OR FETAL OR FETUS* OR FRAGILE X OR GESTATIONAL AGE OR GIRL* OR HYDROPS FETALIS OR INFANCY OR INFANT OR INFANTS OR INFANTILE OR INTRAUTERINE EXPOSURE OR INTRAUTERINE GROWTH OR JUVENILE DERMATOMYOSITIS OR LISSENCEPHALY OR MECONIUM OR MENARCHE)

TI=(MICROPREMIE* OR MOTHERS MILK OR MYELOMENINGOCELE* OR NEONAT* OR NEURAL TUBE DEFECT* OR (NEUROBLASTOMA* NOT CELL*) OR (NEPHROBLASTOMA NOT ADULT*) OR NEURODEVELOPMENT* OR NEWBORN* OR NONIMMUNE HYDROPS OR (OSTEOSARCOMA* NOT (ADULT* OR CELL*)) OR PEDIATR* OR PERINATAL* OR PRADER OR PRENATAL* OR PRESCHOOL OR PRETERM OR PREMATURITY OR PUBERTAL OR PUBERTY OR RETT OR (RHABDOMYOSARCOMA* NOT CELL*) OR (SACROCOCCYGEAL NOT ADULT*) OR SCHOOLCHILDREN OR TODDLERS OR (WILMS NOT ADULT*) OR X LINKED)

### A.3 Biomedical address filter

AD=(MED OR HLTH* OR NEURO* OR PHARM* OR PATHO* OR CLIN OR SURG OR BIOCH*M OR HOSP* OR GENET OR P*EDIAT OR PHYSIO* OR PSYCH* OR MICROBI*L OR (BIOL SAME MOL) OR IMMUN* OR CARDIO* OR CANC OR ONCOL* OR DIS OR BIOMED* OR CELL OR CHILD* OR RADIOL OR EPIDEM* OR BIOTEC* OR HUMAN OR VET OR ANIM OR H*EMATO* OR ANAT OR GYN*ECOL OR DENT OR CARE OR NUTR OR OBSTET OR CEL*ULA*R* OR BIOPHYS)

AD=(ENDOCRIN* OR INFECT OR MED-* OR DERMATOL* OR GASTRO* OR TOXI*OL OR NIH OR BIOSTAT OR AN*ESTHE* OR THERAP* OR HOP-* OR UROL OR KLIN* OR NEPHROL* OR INSERM* OR PREVENT OR OPHTHALMOL OR BIOSCI OR METAB OR ORTHOP*ED OR GENOM* OR TRANSPLANT* OR REPROD OR ORAL OR DIAGNOST OR RH*UMATOL OR BRAIN OR KAROLINSKA* OR VIROL OR DRUG* OR DIABET* OR VASC OR WOMEN* OR CRIT OR VET-* OR NCI OR PULM* OR CHU-* OR OTOLARYNGOL OR HEART)

AD=(ALLERG* OR REHABIL OR POLI*LIN OR PARASITOL OR BIOQUIM OR GERIATR OR HISTO* OR MRC OR RESP OR POPULAT OR BIOENGN OR FAMILY OR GENE OR THORAC OR DISORDER* OR FARMAC* OR EMERGENCY OR HEAD OR MEM-* OR MACROMOL OR NECK OR IRCCS* OR PROT OR EYE OR HOP OR OCCUPAT OR COGNIT OR HYG OR CTR-DIS-CONTROL* OR HYPERTENS OR AGING OR CARDIAC* OR RENAL OR BIOINFORMAT OR CYTO* OR NEONAT*L OR MAXILLOFACIAL OR TUMOR* OR OSPED-* OR LIVER)
